# An artificial intelligence-powered learning health system to improve sepsis detection and quality of care: a before-and-after study

**DOI:** 10.1038/s41746-025-02180-2

**Published:** 2026-01-20

**Authors:** Jérémie Despraz, Raphaël Matusiak, Snežana Nektarijevic, Valerio Rossetti, François Bastardot, Rachid Akrour, Andreas Konasch, Emeline Gauthiez, Olivier Pignolet, Santino Pepe, Jean-Daniel Chiche, Daniel E. Kaufmann, Thierry Calandra, Jean Louis Raisaro, Sylvain Meylan

**Affiliations:** 1 Biomedical Data Science Center, Lausanne University Hospital, Lausanne, Switzerland; 2 Swiss Data Science Center, EPFL, Lausanne, Switzerland; 3 Clinical Informatics Unit, General Directorate, Lausanne University Hospital, Lausanne, Switzerland; 4 Geriatrics Service, Department of Medicine, Lausanne University Hospital, Lausanne, Switzerland; 5 Nursing Directorate, Lausanne University Hospital, Lausanne, Switzerland; 6 Internal Medicine Service, Department of Medicine, Lausanne University hospital, Lausanne, Switzerland; 7 Infectious Diseases Service, Department of Medicine, Lausanne University Hospital, Lausanne, Switzerland; 8 IT Department, Lausanne University Hospital, Lausanne, Switzerland; 9 Medical Directorate, Lausanne University Hospital, Lausanne, Switzerland; 10 Intensive Care Unit, Lausanne University Hospital, Lausanne, Switzerland; 11 Research Centre, Centre Hospitalier de L’ Université de Montréal (CRCHUM) and University of Montreal, Montreal, QC Canada; 12 Immunology and Allergy Service, Department of Medicine and Center for Human Immunology Lausanne and Department of Laboratory Medicine and Pathology, Lausanne University Hospital, Lausanne, Switzerland; 13 Cantonal Doctor Office, Public Health Department, Canton of Vaud, Lausanne, Switzerland; 14 Moorea Hospital, Moorea, French Polynesia; 15 Department of Medicine, CHU LA MILETRIE, Poitiers, France

**Keywords:** Infectious diseases, Health policy, Health services, Data integration, Data processing, Databases, Machine learning, Predictive medicine, Software

## Abstract

Sepsis is a major global health crisis where early recognition and effective management remain significant challenges for healthcare systems. As part of the Lausanne University Hospital sepsis quality of care program, we developed and validated an Artificial Intelligence (AI)-powered Sepsis Learning Health System (SLHS) to enhance sepsis care. The SLHS combines a standardized clinical pathway with HERACLES, an AI algorithm that retrospectively classifies patient data into confirmed, possible, or invalidated sepsis cases every 6 h. Predictions inform dynamic dashboards displaying quality-of-care indicators to guide clinical interventions. Analysis of 97,559 stays in wards using the SLHS and 25,851 stays in control wards showed that in-hospital and 90-day mortality decreased for HERACLES-flagged sepsis in SLHS wards, while control wards did not. Further, sepsis coding increased in SLHS wards but did not change in control wards. This real-world example demonstrates how clinician-integrated AI systems can improve sepsis detection and outcomes.

## Introduction

Sepsis, a life-threatening syndrome caused by a dysregulated host response to infection^1^, is a major global health priority with an estimated 50 million cases and 11 million deaths annually worldwide^2^. Despite major advances in sepsis care, early recognition and effective management remain substantial challenges for healthcare systems^3,4^. The Surviving Sepsis Campaign has established evidence-based guidelines that emphasize timely administration of antibiotics and fluid resuscitation within a comprehensive bundle of interventions as key interventions to optimize patient outcomes^5–7^. However, the implementation, adherence and systematic monitoring of these guidelines remain suboptimal and have predominantly been assessed through retrospective, cross-sectional studies that provide only static snapshots rather than longitudinal insights^8,9^. Moreover, such evaluations frequently rely on International Classification of Diseases (ICD) coding which, despite their widespread adoption, exhibit considerable limitations including low sensitivity and inter-hospital variability. This fundamentally undermines their reliability as sepsis surveillance instruments^10–13^.

The ongoing digital transformation of healthcare infrastructures coupled with the rapid development in Artificial Intelligence (AI) offer unprecedented opportunities to revolutionize healthcare^14^. AI-driven electronic alerting systems and clinical decision support tools have demonstrated promising results in assisting clinicians^15^ in sepsis identification and management protocols^16–19^. However, these solutions frequently lack robust mechanisms for continuous performance evaluation and the capacity for integration of new clinical insights, thereby limiting their real-world effectiveness and sustainable adoption across clinical environments^20–22^.

**Fig. 1:**
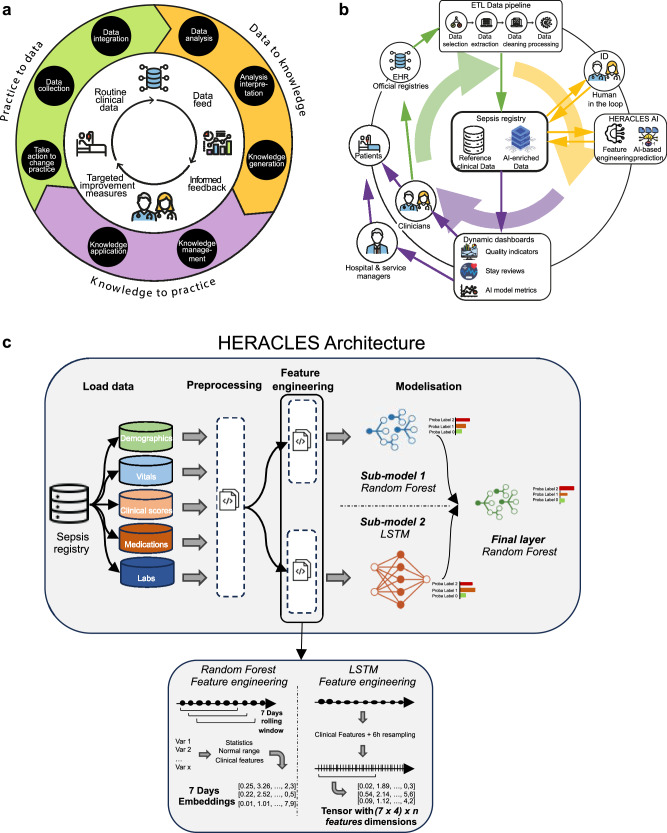
An AI-powered learning health system for sepsis. A learning health system creates a virtuous circle (**a**) where clinical practice generates raw data (green), which is systematically processed into actionable knowledge (yellow). This knowledge is then fed back into clinical practice (purple). The Lausanne sepsis learning health system complements this framework (**b**) through an AI-powered digital pipeline with human oversight. Dashboards inform stakeholders and clinicians with key insights to help improve patient care. Schematic representation of the Health Evaluation and Risk Assessment for Clinical Early Sepsis (HERACLES) prediction model architecture (**c**). Data from the sepsis registry is extracted, pre-processed, and used to compute features for Random Forest and Long Short-Term Memory (LSTM) models. The outputs combine in a final layer that predicts the probability of sepsis for each patient. These predictions enrich the sepsis registry and generate clinical indicators (adapted from Flynn et al.^50^).

Learning Health Systems (LHSs)^23,24^ offer a transformative framework for improving healthcare delivery by creating systematic and continuous feedback loops between clinical practice and research (Fig. 1a). Within this paradigm, routine clinical care generates data which, when adequately analyzed, yields actionable feedback that directly informs and enhances subsequent clinical decision-making and interventions thus enabling healthcare organizations to learn from patient interaction while maintaining the highest standards of care. Yet, transiting from conventional healthcare delivery models toward LHS implementation requires innovation in data integration, workflow optimization, and a sustained commitment to iterative quality improvement methodologies and organized learning, with systematic incorporation of emerging evidence into standardized clinical workflows. Despite the compelling theoretical advantages of this approach, no operational LHS specifically designed for sepsis management has been described to date.

This study fills a substantial gap by describing the first Sepsis Learning Health System (SLHS). The SLHS integrates a standardized clinical pathway (Table 1, Supplementary Fig. 1, and Supplementary Data p. 2-3) with an AI-driven digital monitoring pipeline, fostering continuous quality improvement (Fig. 1). At the core of the monitoring pipeline, a sepsis registry centralizes clinical data of 97,559 patient stays by December 2024. We used it to develop HERACLES (Health Evaluation and Risk Assessment for Clinical Early Sepsis), a Machine Learning algorithm that retrospectively computes sepsis probabilities distribution by classifying 6-h fragments of stays into “no sepsis”, “possible sepsis” or “confirmed sepsis”. HERACLES’ predictions are then used to produce actionable care indicators that enable clinical teams to implement targeted interventions that improve sepsis detection, documentation, treatment adherence, and patient outcomes^25^.

**Table 1:** key element of the standardized sepsis clinical pathway

Key steps	Measures expected
Suspected Sepsis Event	In case of a suspected sepsis event, a clinician prescribes a standardized “sepsis order set” of diagnostic tests through a dedicated form in the electronic health record (EHR), triggering a systematic case review by a medical member of the sepsis team (see Supplementary Data p. 2).
Additional Case Review	Sepsis cases without an order set, identified within the framework of Infectious Disease (ID) consultations, can also be reviewed by the sepsis team.
Expert Evaluation	Each case is evaluated by combining the clinical judgment of an ID specialist with a sequential (or sepsis-related) organ failure assessment (SOFA) score increase of ≥2 points according to the Sepsis-3 Consensus Criteria. Further details on its calculation are provided in the Supplementary Data p. 12–15.
Case Classification	Based on this review, and according to Sepsis-3 Consensus Criteria, cases are assigned to one of three mutually exclusive labels: “no evidence of sepsis”, “possible sepsis” or “confirmed sepsis” (see Table 4). These labels are further differentiated according to sepsis and septic shock for “confirmed sepsis” and “possible sepsis” according to missing information vs. difficulty to establish causality between (suspected) infection and organ dysfunction (see Table 4 for detailed definitions).
Documentation	All suspected sepsis events, regardless of their classification are documented using a dedicated form that was introduced into the hospital’s EHR as part of the Sepsis Program. The content of the form and the sepsis onset time (time 0—see Supplementary Data p. 2-3) are directly extracted and uploaded into the sepsis-dedicated registry.

## Results

The primary objective of this study was to develop and validate the SLHS on primary outcomes to support the Lausanne University Hospital (CHUV) “Sepsis Quality of Care Program”, a major initiative within the hospital’s 5-year strategic plan.

### Implementation of the sepsis learning health system and study population

**Fig. 2:**
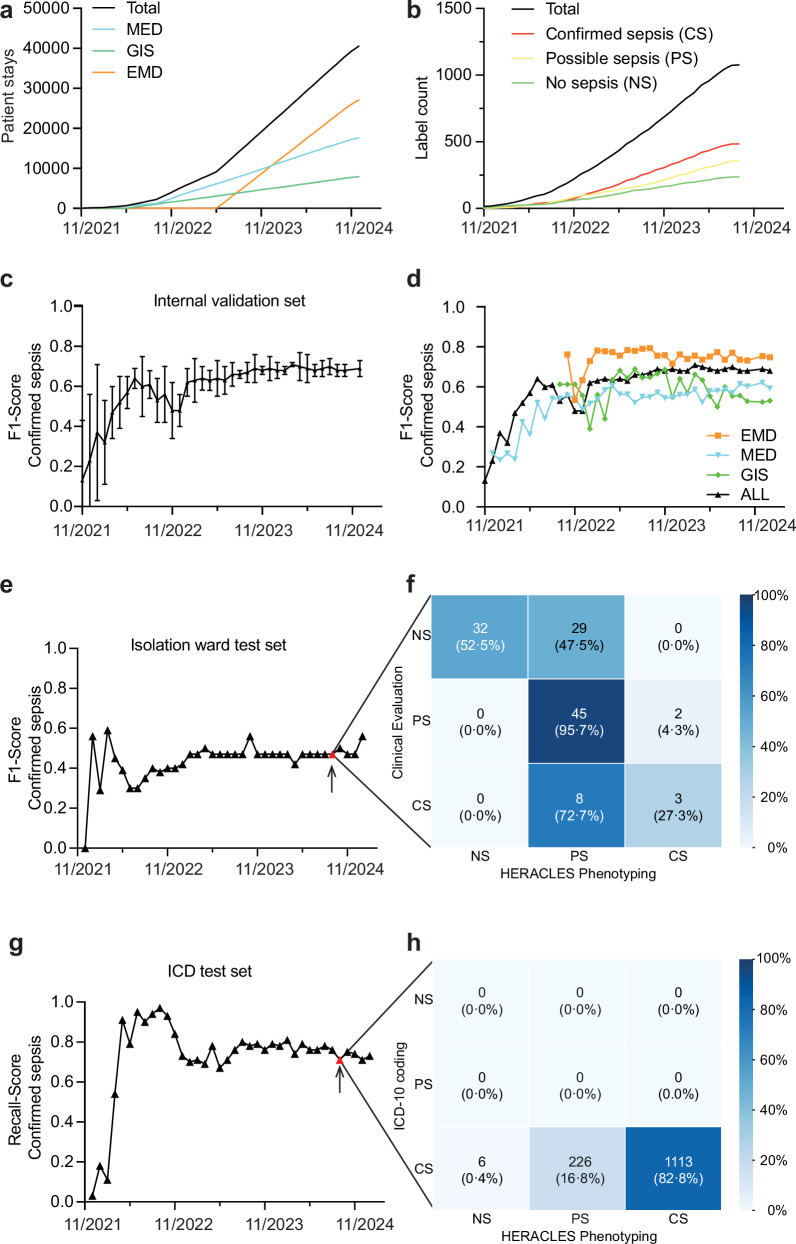
Deployment of the sepsis learning health system. Patient accumulation after deployment in program wards (**a**) medicine department (MED, blue), gastrointestinal surgery (GIS, green), emergency department (EMD, orange), and cumulative total (black). Accumulation of sepsis labels over time (**b**) no sepsis (green), possible sepsis (yellow) and confirmed sepsis (red) and total (black). Health Evaluation and Risk Assessment for Clinical Early Sepsis (HERACLES) performance for confirmed sepsis recognition expressed as F1-score and AUROC. Overall performance over time (**c**) and context-specific performance (**d**)—IE training for GIS (green), MED (blue), and EMD (orange) using department-specific events, compared to overall performance (black). Additional validations include: (i) a 6-month fully annotated cohort of the isolation ward (**e**) with confusion matrix for August 31, 2024, HERACLES version (**f**) for no sepsis (NS), possible sepsis (PS) and confirmed sepsis (CS), and (ii) 2022–2023 ICD-10 sepsis-coded hospital stay (Supplementary Table 1) (**g**) with confusion matrix from August 31, 2024 HERACLES version (**h**).

We constructed and progressively deployed the SLHS from January 1, 2020 onward. By December 31, 2024, the SLHS registry included 97,559 hospital stays across 63 hospital units, representing 57,180 unique patients (Table 2, Supplementary Tables 2–3). As comparators, we used 25,851 stays in 126 control wards including Locomotor Apparatus, Cardiovascular, Neurosciences and Oncology Departments’ (LAD, CVD, NSD, and OD) wards (Table 2). The SLHS clinical pathway was deployed in four phases as outlined in Methods, beginning on November 1, 2021, in the infectious disease (ID) service’s isolation ward (where hematological malignancy patients are admitted). This was followed by a pilot in two additional Medicine Department (MED) wards on March 1, 2022, and a full rollout by November 01, 2022. Further deployments included Gastrointestinal Surgery (GIS) wards on June 1, 2022, and the Emergency Department (EMD) on June 1, 2023, ultimately covering 40,502 of the 97,559 patient stays across all participating wards by December 31, 2024 (Fig. 2a and Table 2).

**Table 2:** Demographics of patient cohorts

	Extended SLHS wards cohort	Limited SLHS wards cohort	Control (non-SLHS) wards
	Hospital stays including transit in a SHLS ward (EMD, MED, GIS)	Hospital stays limited to SHLS wards (EMD, MED, GIS)	Stays in selected control wards, excluding transit in SHLS wards (LAD, OD, CVD, NSD)
	total	total	total
Demographics			
Patients	57,180	36,825	16,030
Stays	97,559	61,129	25,851
Age (median, range or IQ)	68.0 [52.0–79.0]	68.0 [51.0–81.0]	65.0 [55.0–75.0]
Male	52.9%	50.4%	59.8%
Female	47.1%	49.6%	40.2%
Length of stay (days, range or IQ)	7.0 [3.0–14.0]	5.0 [1.0–10.0]	4.0 [2.0–10.0]
In-hospital mortality	0.04%	0.03%	0.01%
Comorbidities			
Charlson Comorbidity index (median points, range or IQ)	1.0 [0.0–3.0]	1.0 [0.0–3.0]	2.0 [0.0–3.0]
Myocardial Infarction (%)	7.57	4.63	11.1
Congestive Heart Failure (%)	13.22	13.23	14.77
Peripheral Vascular Disease (%)	5.26	3.84	18.28
Cerebrovascular Diseases (%)	6.97	2.15	8.1
Dementia (%)	11.69	13.18	2.79
Chronic Pulmonary Disease (%)	10.89	12.38	6.55
Rheumatic Disease (%)	1.88	1.81	1.37
Peptic Ulcer Disease (%)	1.10	1.14	0.24
Diabetes without Chronic Complications (%)	11.41	10.56	12.15
Diabetes with Chronic Complications (%)	2.74	2.94	2.47
Renal Disease Mild to Moderate (%)	13.58	14.60	11.74
Severe Renal Disease (%)	6,09	6,61	6.62
Mild Liver Disease (%)	4.31	4.51	3.75
Moderate or Severe Liver Disease (%)	1.48	1.46	0.48
Any Malignancy (%)	13.29	15.03	20.65
Metastatic Solid Tumor (%)	8.22	8.80	11.36
HIV Infection no AIDS (%)	0.05	0.06	0.03
HIV and Opportunistic Infection (%)	3.38	2.91	1.44

Opportunistic diseases include Candidiasis of esophagus, coccidioidomycosis, Cryptococcosis, chronic intestinal cryptosporidiosis, cytomegalovirus disease, HIV-related encephalopathy, herpes simplex esophagitis, histoplasmosis, chronic intestinal isosporiasis, Kaposi’s sarcoma, Lymphoma, multiple forms, Mycobacterium avium complex infection, Tuberculosis, Pneumocystis carinii pneumonia, Salmonella sepsis, Toxoplasmosis of brain, HIV wasting syndrome.

*AIDS* acquired immunodeficiency syndrome, *CVD* cardiovascular department, *EMD* emergency department, *GIS* gastrointestinal surgery, *HIV* human immunodeficiency virus, *IQ* interquartile, *MED* medicine department, *NSD* neurosciences department, *LAD* locomotor apparatus department, *OD* Oncology department, *SLHS* sepsis learning health system.

By August 31, 2024, a total of 1043 suspected sepsis events were documented across 961 hospital stays using (Table 3) the standardized sepsis clinical pathway (see Supplementary Material). Upon assessment by ID specialists, 230 cases (22.0%) were classified as no infection or infection without sepsis, 341 cases (32.7%) as possible sepsis and 472 cases (45.2%) as confirmed sepsis including septic shock (Fig. 2b, Table 4). Confirmed sepsis cases were distributed across the MED wards (39.0%), EMD (30.0%), GIS (11.5%), and other services (19.5%). Among documented cases, 69.3% of confirmed sepsis cases occurred in male patients. Patients with confirmed sepsis were generally older (median age: 69 years) compared to those with possible sepsis (67 years) and no sepsis cases (63 years). In-hospital mortality was 5.7% for no sepsis cases, 11.1% for possible sepsis cases, and 13.3% for confirmed sepsis cases. Among confirmed sepsis cases, 83.3% were linked to an ICD-10 code for sepsis, while 35.5% of possible sepsis cases had a sepsis-related ICD code, suggesting potential under-documentation of sepsis cases. We note that these coded cases do not represent the total number of sepsis cases within these wards. HERACLES is designed to complement these cases through inference.

**Table 3:** Patient characteristics in SLHS wards

	HERACLES training/cross validation cohort				Test set 1 Isolation Ward	Test set 2 ICD-10 Sepsis Codes^a^
Modeling						
Services	All, GIS, EMD, and Other	MED only	GIS only	EMD only	MED only	All, GIS, EMD, and Other
Demographics						
Number of sepsis events	1043	407	118	316	11	1345
Number of stays	961	371	106	289	119	1345
Number of patients	906	342	99	276	83	1180
Male	575 (63.5%)	217 (63.5%)	59 (60.0%)	185 (67.0%)	49 (60.0%)	720 (61.0%)
Female	331 (36.5%)	125 (36.5%)	40 (40.0%)	91 (33.0%)	34 (40.0%)	460 (39.0%)
In-hospital mortality	105 (10.1%)	50 (12.2%)	5 (4.2%)	25 (7.9%)	2 (18.2%)	290 (21.6%)

*EMD* emergency department, *GIS* gastrointestinal surgery, *HERACLES* health evaluation and risk assessment for clinical early sepsis, *ICD* international classification of diseases, *MED* medicine department.

^a^See Supplementary Table 1.

**Table 4:** Definitions of HERACLES training labels

Term	condition	label	definition
No sepsis	No infection	0	No Infection
	Infection	0	Infection without organ failure or delta sequential (or sepsis-related) organ failure assessment (SOFA) less than 2 points
Possible sepsis	Sepsis-like	1(a)	Infection with organ failure and/or delta SOFA less than 2 points or missing SOFA information
	Sepsis sensu lato	1(b)	Infection with organ failure and/or delta SOFA equal or greater than 2 points (with alternative etiology for organ dysfunction)
Sepsis	Sepsis sensu stricto	2(a)	Infection with organ failure and delta SOFA equal or greater than 2 points. (no alternative aetiology for organ dysfunction)
	Septic shock	2(b)	Sepsis sensu stricto AND vasopressor AND serum lactate greater than 2.0 mmol/L

These labels are informed by current Sepsis-3 consensus definitions.

*SOFA* sequential (or sepsis-related) organ failure assessment.

### AI-based sepsis identification using HERACLES

The primary objective of HERACLES was to automatically deliver accurate and comprehensive sepsis recognition post-discharge for patients to generate performance indicators (see Supplementary Movie). Following the sepsis care program rollout, HERACLES was continuously trained on the SLHS registry growing cohort of annotated sepsis cases. Its F1 score for confirmed sepsis improved over time, reaching 0.71, before stabilizing at 0.68 (Fig. 2c), with a precision of 0.76, recall of 0.62, and an Area Under the Receiver Operating Characteristic (AUROC) of 0.88 for confirmed sepsis vs. no sepsis cases and 0.81 for confirmed and possible sepsis vs. no sepsis cases (Supplementary Table 4) using internal validation.

To investigate this performance plateau, we trained service-specific versions of HERACLES for MED, GIS, and EMD wards (Fig. 2d). However, these specialized models did not substantially outperform the general model, yielding F1 scores and AUROCs of 0.57 and 0.85 (MED) and 0.55 and 0.86 (GIS), respectively for confirmed sepsis. Only the EMD-specific model outperformed the general model in predicting confirmed sepsis cases with F1 score and AUROC of 0.77 and 0.88 (Fig. 2d and Supplementary Table 4). Further analysis revealed that 50% and 82% of HERACLES-flagged sepsis cases occurred within the first 48 h and 96 h of admission respectively (Supplementary Fig. 2), indicating that most predicted cases are consistent with community-acquired sepsis.

On the first test dataset, consisting of 83 fully annotated patients (119 stays) admitted to the isolation ward during a 6-month period in 2020, HERACLES’ recall for confirmed sepsis cases was 0.27, with a precision of 0.60, and F1 score of 0.37, while the mean F1 score was 0.37 (Fig. 2e, f and Supplementary Table 4). However, when possible and confirmed sepsis cases were combined, HERACLES demonstrated higher precision, reinforcing its tendency to flag possible sepsis rather than dismissing cases entirely. The use of an ensemble method rather than Long Short-Term Memory (LSTM) or Random Forest (RF) alone improved the F1 score and HERACLES’ performance (Supplementary Fig. 3 a and b). When evaluated against the ICD-10-based test dataset (1345 sepsis-coded cases from 2022 to 2023), HERACLES demonstrated a recall of 0.83 for confirmed sepsis, identifying 1113 of 1345 cases. Notably, the 226 misclassified cases were predicted as possible sepsis, but very few (6 out of 1345) were classified as no sepsis, indicating again a strong tendency toward flagging possible sepsis cases but not toward a full misclassification as no sepsis (Fig. 2g, h).

### Estimating SLHS’s global impact on sepsis care and patient outcomes

**Fig. 3:**
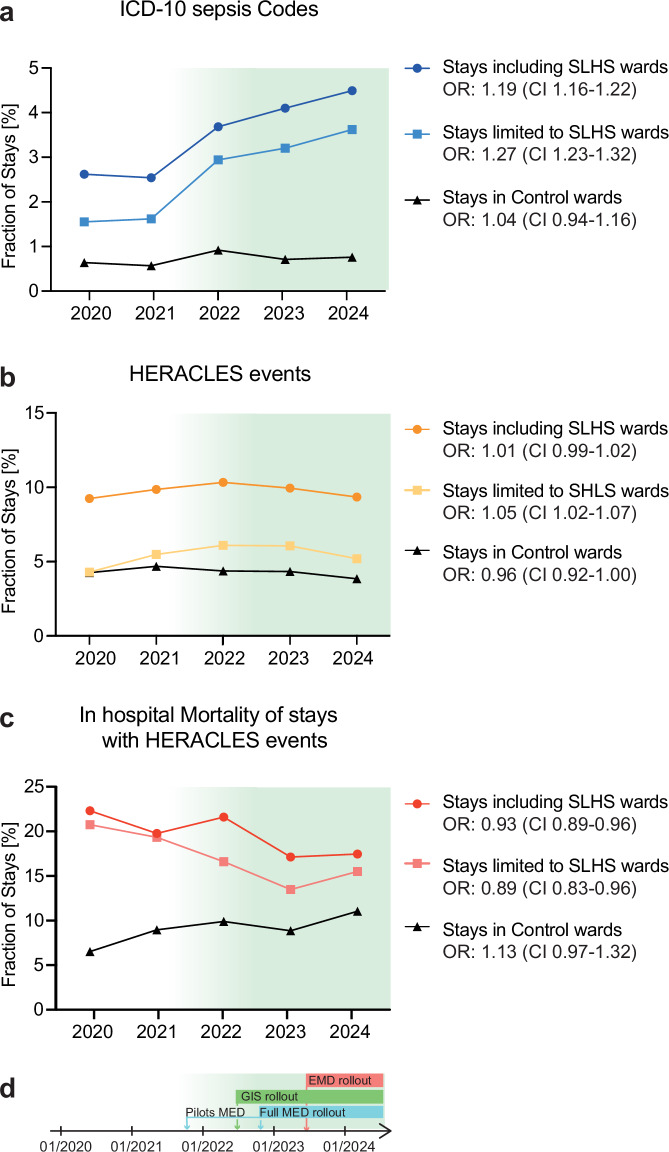
SLHS impact on documentation and mortality. Percentage of patients with sepsis-specific ICD-10 codes (**a**) in stays including program wards (dark blue), limited to program wards (light blue), and control wards (black). Stays including SLHS wards: patient stay during which the patient spent any portion of their stay in at least one ward where the SLHS was deployed. Stays limited to SLHS wards: patient stays confined entirely to wards where the SLHS was deployed. Percentage of patients with high HERACLES sepsis probability (**b**) in stays including program wards (dark orange), limited to program wards only (light orange), and control wards (black). In-hospital mortality (**c**) per year in patients with high HERACLES sepsis probability in stay including program wards (dark red), limited to program wards only (light red), and compared to control wards (black). SLHS rollout timelines (**d**). ORs (odds ratio) reflect the contribution of the year on the outcome in a logistic regression (LR) model.

SLHS deployment was associated with substantial improvement in sepsis documentation. ICD-10 sepsis coding increased in statistically significant proportions in both settings where SLHS was implemented: from 2.62% to 4.48% (odds ratio (OR): 1.19, 95% confidence interval (CI) 1.16–1.22) for all stays including SLHS wards (see definitions in methods), and from 1.55 to 3.58% (OR: 1.27 (CI 1.23–1.32)) for stays exclusively within SLHS wards (Fig. 3a). Control wards without SLHS implementation showed no significant change in sepsis coding (0.64–0.78% OR: 1.04 (CI: 0.94–1.16)). In comparison, the proportion of HERACLES-flagged sepsis cases, used as a proxy for the number of sepsis events, remained stable across stays including SLHS wards (9.23–9.24%, OR: 1.01 (CI 0.99–1.02)), control wards (4.24–3.67% OR: 1.05 (CI: 1.02–1.07)) but not for stays limited to SLHS wards (4.28–5.16%, OR: 1.05 (CI 1.02–1.07)) (Fig. 3b and Supplementary Tables 5 and 6).

Patient outcomes also improved following SLHS implementation. For HERACLES-flagged sepsis cases, a decrease in in-hospital mortality reached statistical significance in stays limited to SLHS wards (20.54% to 15.27% OR: 0.89 (CI: 0.83–0.96)) and stays including SLHS wards (22.21% and 17.76% OR: 0.93 (CI: 0.89–0.96)) (Fig. 3c). Similarly, the decrease in 90-day mortality reached statistical significance for patients with stays limited to SLHS wards (32.99–26.11% OR: 0.91 (CI: 0.86–0.97)) and stays including SLHS wards (29.75–25.01%; OR: 0.94 (CI: 0.91–0.97)). In contrast, control wards showed concerning trends, albeit not reaching statistical significance, with in-hospital mortality for patient stays with HERACLES-flags increasing from 6.53% to 11.74% (OR: 1.13 (CI: 0.97–1.32)) and 90-day mortality remaining unchanged (12.56–15.02%; OR: 1.04 (CI: 0.92–1.19)) (Fig. 3c and Supplementary Tables 8 and 10). Furthermore, mortality in the SLHS wards for patients without sepsis flags showed a non-significant reduction in both in-hospital mortality (0.86–0.91% (OR: 0.98 CI: 0.91–1.05)) and 90-day mortality (6.15–6.41%, (OR: 0.98, CI: 0.95–1.01)). For patients with stays including SLHS wards, in-hospital mortality remained stable (1.21–1.41%, (OR: 1.01, CI: 0.96–1.06)) while 90-day mortality showed a significant decrease (6.46–6.34%, (OR: 0.97, CI: 0.95–0.99)). Control wards exhibited a stable trend in in-hospital mortality (0.43–0.42%, (OR: 1.02, CI: 0.86–1.21)) but a significant decrease in 90-day mortality (4.07–3.18%, (OR: 0.91, CI: 0.86–0.97)) (Supplementary Table 9 and 11). Taken together, these data are consistent with a reduction in mortality in sepsis stays amongst a decreasing mortality trend in SLHS wards contrasting with a stable situation in control wards. To further understand the impact of the learning system, we looked at time-to-antibiotics as a performance indicator. We leveraged the cases manually reviewed within the program as part of the antimicrobial stewardship form review (Table 1, Supplementary Material, and Supplementary Tables 13–15 and 16–18), which includes the manual annotation of t_0_ (see Supplementary Data p. 2–3). This enables the accurate computation of time-to-antibiotics for a subset of patients for whom we observed that adherence to the sepsis pathway guidelines is significantly associated with timely antibiotic administration for confirmed sepsis cases. These patients, when treated according to the pathway guidelines, are significantly more likely to receive antibiotics within 1 h after t_0_ than those would are not (*p* < 0.001, Supplementary Table 14). This association remains statistically significant when considering antibiotic administration within 3 h after t_0_ (*p* = 0.042, Supplementary Table 15). Of note, the SLHS dashboard continuously displays these updated performance and outcome indicators (see Supplementary Movie).

## Discussion

In this study, we have successfully developed, validated and implemented a SLHS within our clinical setting. This innovative system integrates a standardized sepsis clinical pathway with an AI-powered digital data pipeline, resulting in demonstrable improvement in sepsis recognition, management, and documentation. The multifaceted approach combined three key components: structured manual documentation through a standardized sepsis clinical pathway, a robust automated sepsis registry, and HERACLES, our novel AI-based sepsis phenotyping algorithm, interfaced through dashboards. These elements create a dynamic, continuously evolving framework that drives quality improvement in sepsis care by enabling real-time performance feedback and iterative refinement of clinical processes.

The decrease in both in-hospital and 90-day mortality as well as improved time-to-antibiotics indicator are consistent with an improvement of sepsis care through the deployment of our learning health system. The SLHS strengthens adherence to evidence-based best practices through its feedback mechanisms, allowing clinical teams to identify areas of improvement and implement targeted interventions. Perhaps most importantly, the system substantially enhances the ability to detect sepsis cases that would otherwise remain unrecognized or undocumented in conventional care models.

HERACLES serves as a cornerstone of our learning health system by providing automated sepsis probability estimates that complement traditional clinical documentation. While ICD-10-based sepsis coding suffers from substantial underreporting^26–29^, HERACLES consistently identified sepsis in 9–10% of hospital stays across all SLHS wards and 5% specifically within the SLHS EMD, MED, and GIS wards, compared to just 2–4% captured by ICD-10 codes. These results align with work by Rhee et al. reporting 5.99% sepsis incidence using a rule-based approach compared with 2.5% by explicit coding^29^. Although our study focused on three departments (MED, GIS, and EMD), these represent over half of the hospital’s adult medicine capacity. Prior research indicates that over 85% of sepsis cases originate outside of Intensive Care Units (ICU), with the EMD identifying 80% of initial cases^30–32^. Consistent with established literature, HERACLES detected sepsis primarily during the first days of hospitalization, validating its utility as a reliable proxy for institutional sepsis incidence.

HERACLES achieved AUROCs comparable to or exceeding current state-of-the art AI-based sepsis detection models (0.88 for confirmed sepsis vs. no sepsis and 0.81 for confirmed and possible sepsis vs. no sepsis), with strong recall and precision for confirmed sepsis cases (Supplementary Fig. 4). Unlike many prior studies that relied on ICD-10 codes as ground truth, SLHS offers a timing of events and incorporates clinician-labeled cases, providing a more accurate training dataset^26–29^.

As a fully automated tool that continuously retrains, HERACLES supports clinical documentation while strengthening our institutional Sepsis Program’s adherence to Centers for Disease Control and Prevention (CDC)‘s Sepsis Core Elements^33^ by enhancing oversight and feedback mechanisms critical to the learning system.

Despite its strengths, HERACLES experienced performance variations during institutional expansion that possibly reflect variations in patient profiles, underlying medical conditions, and differences in clinical workflows across newly integrated wards^34–36^. The F1 score initially improved but plateaued following deployment in GIS and EMD units. Specialized models trained for specific departments showed mixed results; the EMD-specific model achieved an F1 score of 0.77 for confirmed sepsis cases, while MED and GIS models did not outperform the general model. Given sepsis heterogeneity^37^, future refinements could explore endotype-based modeling approaches for more personalized detection strategies^34–36,38,39^.

The SLHS provides our hospital’s Quality and Security Governance with continuous ward level monitoring capabilities enabling targeted quality improvement interventions. It enables the documentation of improved clinical outcomes specifically in program wards with reduced in-hospital and 90-day mortality. The observed increase in sepsis ICD-10 coding rates from 2022 to 2023 would represent an additional 250,000 Swiss francs (modelling based on prior institutional data) with potential 5-year recovery approaching one million Swiss francs yearly (comparing 2020, 2021 to 2023, 2024). This figure could further increase through systematic review of cases where HERACLES indicates high sepsis probability, demonstrating the financial value of AI-assisted sepsis detection beyond clinical benefit.

Our study provides the first institutional implementation of a validated AI-powered SLHS. Its prospective design with 97,559 patient stays ensures real-world applicability, while multiple independent test sets enhance evaluation robustness. The system offers a scalable framework potentially applicable to other acute conditions including stroke, myocardial infarction, and thrombosis.

Several limitations warrant acknowledgment. First, the impact of HERACLES on patient-centered outcomes could not be properly controlled as control wards contained patients with different pathologies than those in the SLHS wards. Institutional constraints only permitted us to run an evaluation through a quasi-experimental study. Further, HERACLES currently operates retrospectively, predicting sepsis post-discharge rather than in real-time. Such a transition will require additional legal, ethical, and technical considerations, including real-time data access, integrity protocols, and external validation of a monocentric study. Our algorithm is also limited by its 6 h-window. This limits the ability to develop performance indicators such as time-to-antibiotics administration metrics and represents another limitation requiring future refinement. As a result, while HERACLES t_0_ is left for future work, time-to-antibiotics indicators can only be computed through a manual review, risking selection bias. Currently deployed in MED, GIS, and EMD settings, planned expansion to ICU is forthcoming. Finally, the system’s long-term sustainability depends on a “human-in-the-loop” approach, where expert validation continues to refine AI predictions.

The SLHS demonstrates the potential of integrated AI solutions to create self-improving healthcare systems that enhance sepsis recognition, monitoring and management. By establishing a continuous feedback loop between clinical practice and data-driven insights, this approach provides a blueprint for addressing complex healthcare challenges while improving patient outcomes and institutional efficiencies beyond sepsis.

## Methods

### Study design

The SLHS combines a standardized sepsis clinical pathway with a digital monitoring system that tracks vital and biological parameters, and quality indicators. It offers near real-time feedback, allowing clinical teams to improve practices and evaluate the pathway’s effectiveness. Figure 1a gives an overview of the SLHS. This prospective, observational, quasi-experimental, single-center cohort study leveraged electronic health records (EHR) and administrative data from the CHUV, a tertiary 1100-bed care center in Switzerland with an average yearly flow of 30,000 patients for 56,000 hospital stays. The study included adult patients (≥18-year-old) admitted in the EMD, MED, and GIS wards. COVID-19 wards were excluded to minimize COVID-19 impact on our analysis. Eligible patients were those admitted on or after January 1, 2020, with a completed hospital stay by December 31, 2024. Following the preparation, testing and deployment of the program, the study comprised four periods/phases: (1) a pre-deployment phase from January 1, 2020 to October 31, 2021, (2) a pilot pathway deployment phase from November 1, 2021 to May 30, 2022; (3) a sequential deployment phase from June 1, 2022 to August 31, 2023 (deployment of GIS wards on June 1, 2022, MED wards on October 1, 2022, and EMD on June 1, 2023), and (4) a maintenance phase from September 1, 2023 to December 31, 2024. The program wards were paired with similar wards (see below in SLHS evaluation) for outcomes comparison. Time-to-antibiotics was assessed through a dedicated analysis of patients identified by the use of the program’s orderset (pathway use cohort) or via the Infectious Disease consulting service (controls). Control cases were reviewed in identical fashion (i.e., with the antimicrobial stewardship form, see “additional case review” in Table 1 and Supplementary Material) to those in the context of the sepsis program, enabling precise calculation of time-to-antibiotics. We then compared the distribution of antibiotic administration times between patients managed according to the sepsis pathway guidelines and those who were not.

### Standardized sepsis clinical pathway

The goal of the standardized clinical pathway (see Supplementary Data pp. 2 and Supplementary Fig. 1) is to provide healthcare workers (nurses and physicians) with a structured, evidence-based approach that leads them through guidelines for early detection, diagnosis, and rapid management of sepsis cases. The pathway consists of several steps that involve clinicians in the wards and experts from the Program’s “sepsis team”, which consists of a registered nurse, an infectious diseases physician, and a data scientist as outlined in Table 1.

The pathway was deployed across several wards in a phased manner, starting on November 1, 2021, in the isolation ward of the MED, followed on 01.03.2022 by a pilot in 2 of the 34 MED wards (pilot pathway deployment phase). The sequential deployment phase included the GIS wards (01.06.2022), the completion of the MED wards roll out (01.10.2022) and the EMD (01.06.2023), gradually covering a total of 63 wards. Nursing and medical teams on wards were trained prior to deployment and received additional training biannually at ward level with morbidity and mortality rounds based on cases identified by the sepsis team including a program refresher. The sepsis team briefed the hospital directorate on a bi-yearly basis. Staff in control wards did not receive any training. Patient stays are separated into: “Stays including SLHS wards” which consist of patient stay during which the patient spent any portion of their stay in at least one ward where the SLHS was deployed, and “stays limited to SLHS wards” which consist of patient stays confined entirely to wards where the SLHS was deployed.

### SLHS monitoring pipeline

To systematically assess the impact of the standardized clinical pathway on sepsis-related outcomes, we developed and implemented an automated digital monitoring pipeline (Fig. 1b), built on three key components:

*Sepsis Registry*: A dedicated registry that combines a relational database optimized via a star-schema model^40^ with a knowledge graph for semantic enrichment, aligned with the Swiss Personalized Health Network (SPHN) and Findability, Accessibility, Interoperability, and Reuse (FAIR) data principles^41–47^. It integrates terminologies like the Systematized Nomenclature of Medicine - Clinical Terms (SNOMED_CT) and Logical Observation Identifiers Names & Codes (LOINC). Data is integrated daily from the Clinical Data Warehouse through automated Extract, Transform, Load (ETL) processes, enriched with computed clinical parameters (e.g., National Early Warning Score (NEWS) and Sequential or Sepsis-related Organ Failure Assessment (SOFA) scores) and real-time quality indicators (see Supplementary Data pp. 4–15). Processes are containerized (Docker) and automated (Jenkins).

*An AI-based sepsis phenotyping algorithm*: HERACLES retrospectively computes sepsis probabilities distribution of sepsis labels (i.e., no sepsis, possible sepsis, confirmed sepsis) every 6 hours (see Table 4), identifying documented and potentially undocumented cases for expert validation and registry inclusion. The 6-hours segment is a compromise between data completeness (laboratory values, vital signs), particularly outside of ICU, and computation resources for the daily update of the database. HERACLES integrates ensemble learning, combining Random Forest (RF)^48^ and Long Short-Term Memory (LSTM)^49^ models (see Fig. 1c, Supplementary Data pp. 16–18).

The RF model processes 240 clinical features aggregated over 7-days rolling windows, with embeddings labeled based on the most severe condition documented in the standardized clinical pathway EHR form. Only embeddings overlapping documented sepsis onset are labeled positively, others are labeled as no sepsis. The LSTM model operates on sequential data resampled at 6-hours intervals, receiving inputs as tensors representing seven consecutive days. It similarly assigns the most severe observed condition as the target label within each window. HERACLES’s final prediction integrates the outputs of the RF and LSTM models through an additional RF layer, which takes as input the class probabilities predicted by each sub-model (three from RF and three from LSTM, resulting in six features). This ensemble generates robust sepsis probability predictions every 6 hours. Further details on feature engineering, model training, handling missing data, and consistency checks are provided in the Supplementary Data (pp. 16–18).

*User-Centric Dashboards*: Outputs from HERACLES, real-time quality indicators, and clinical patient data are displayed through interactive dashboards. Developed iteratively with clinician feedback, dashboards update daily, providing immediate actionable insights (Supplementary Movie).

### Statistical analysis

*HERACLES performance evaluation*: To ensure continuous improvement and robustness, HERACLES is trained and internally validated over time using 5-fold cross-validation. We compared the model’s output probabilities against ground truth labels to compute precision, recall, F1-score metrics, and AUROC. To obtain one single label per stay, sepsis predictions were generated every 6 hours and aggregated by selecting the highest recorded sepsis probability. To balance precision with recall, we calibrated and optimized the model’s threshold to ensure a minimum recall of 0.7 for confirmed sepsis cases in the training set, hence enhancing the detection of confirmed sepsis and septic shock. The training and validation procedure was conducted every month using manually labeled past and new sepsis cases from the registry. After validation, each new model was deployed in production if its measured performance was better than the previous one.

In addition to internal validation, we performed an external validation on two independent test datasets that were neither used for training nor included in the registry. The first test set consists of 83 patients (119 hospital stays) in 2020 from the Isolation ward of the ID service where hematological malignancy patients are admitted. To ensure exhaustive representation, an ID specialist conducted a 6-month review of all patient stays, assigning 61, 47, and 11 labels for no sepsis, possible sepsis, and confirmed sepsis, respectively. We then counter-validated these assignments with a second ID specialist and an internal medicine specialist, quantifying inter-rater agreement (kappa = 0.83 (0.67–0.99)). In case of disagreement, the case was reviewed jointly for consensus. The second test set consists of only confirmed sepsis and septic shock patient stays identified using sepsis ICD-10 codes (Supplementary Table 1). We identified 1345 hospital stays with sepsis ICD-10 codes from 2022 to 2023, ensuring that none overlapped with cases documented through the standardized sepsis clinical pathway, allowing us to independently evaluate HERACLES’ performance.

*SLHS evaluation*: To assess the impact of SLHS on sepsis care, we compared primary outcomes for the entire population—mortality (in-hospital, 30-day, and 90-day) and sepsis documentation (ICD coding, see Supplementary Table 1)—before and after deployment. Control wards included LAD, CVD, NSD and OD. For time-to-antibiotics, we compared selected patients treated according to pathway guidelines vs. Controls (see Table 1 and Supplementary Material). We applied a logistic regression to assess the impact of the program on categorical outcomes, such as number of ICD-coded stays, in-hospital mortality, and HERACLES flagged stays for the period 2020–2024. The regression parameters included year of admission, sex, age, and Charlson comorbidity index to account for potential confounders. Statistical significance was assessed through the OR and CI of the regression parameters. For time-to-antibiotics outcomes, we used propensity score matching (correcting for age, sex, and Charlson comorbidity index) with Chi-squared statistical tests.

### Large language model use

We used a large language model for language optimization.

### Ethics approval

The Legal Affairs Unit of CHUV confirmed that this study falls outside the scope of the Swiss research legislation (Federal Act on Research involving Human Beings, Human Research Act, HRA, SR 810.30) and therefore does not require ethics committee authorization, in accordance with Swiss legislation and institutional guidelines, thus waiving the need for informed consent.

## Supplementary information

Supplementary Materials

Supplementary Movie

## Data Availability

The datasets generated and/or analyzed during the current study are not publicly available due to legal constraints (consent waived as quality of care) but are available from the corresponding author on reasonable request.

## Code availability

The underlying code for this study [and training/validation datasets] is not publicly available for proprietary reasons.
