# Supplementary material for: An artificial intelligence-powered learning health system to improve sepsis detection and quality of care: a before-and-after study
Source: NPJ Digit Med. 2026 Jan 20;9:106. doi: 10.1038/s41746-025-02180-2 (PMC12864897; doi:10.1038/s41746-025-02180-2)
Supplement: Supplementary file 1 — Supplementary Materials [file 41746_2025_2180_MOESM1_ESM.docx]

**Supplementary data**

Contents:

1. The Sepsis care program at CHUV 2
2. Variables used in the pipeline 3
3. Score calculations 4
   1. NEWS computation 4
   2. SOFA score computation 7
4. Modelisation 16
5. Dashboards 19
6. References 20
7. Supplementary tables 21
8. Supplementary figures 45
9. Abbreviations 49

#

# **1. The Sepsis Care Program at CHUV**

Clinical pathway:

We constructed a program articulated around a care pathway (Supplementary Fig. 1) designed by integrating key elements of the 2021 draft of the SSC and an analytical pipeline. Sepsis screening is based on clinical suspicion or an elevated National Early Warning Score (NEWS) (≥ 5 points) [1] combined with a suspected infection [2]. To assist clinical teams, the computation of the NEWS and Sequential Organ Failure Assessment (SOFA) [3] score is automated and directly embedded in the existing EHR infrastructure together with an alert system to help promptly identify a patient's clinical deterioration. The results of these computations are automatically stored in the patient records and displayed as a graph together with the patient's vitals to allow clinicians to track their evolution over time. Entry in the sepsis pathway includes clinical evaluation with a qSOFA^[[1]](#footnote-1)^ (quick SOFA score) [4] and a “sepsis order set”, comprising basic lab works (lactate, SOFA score components, blood cultures and procalcitonin). The purpose of this order set is to maximize the completeness of physiological parameters related to sepsis. More specifically, this lab order set is implemented in the EHR and its utilization automatically triggers an acceleration of the SOFA score computation and is used to identify suspected sepsis cases in patients’ EHR, enabling retrospective expert reviews.

Following the SSC's recommendation for antibiotic reassessment, a rapid feedback mechanism after 48-72 hours following the prescription of a sepsis order set was implemented to curb potential antibiotic overuse or restrict antibiotic spectrum [5]. We created for this purpose a clinical form triggered by the sepsis orderset (the antimicrobial stewardship form) for ID specialists to annotate sepsis events with specific information, including event categorization, onset of initial symptoms, adherence to sepsis guidelines and time zero (t_0_). Additionally, sepsis cases identified by the infectious disease service and not managed via the recommended pathway are also documented by an ID specialist for these criteria. For t_0_, context was considered. In cases within the first 48 hours of admission (IE community-acquired sepsis), t_0_ was constrained to check in time in the Emergency Department. In cases beyond the first 48 hours of admission (IE nosocomial sepsis), t_0_ was aligned with first clinical sign of deterioration as quantified by NEWS ≥ 5points or if, occuring prior, the first sign of clinical suspicion of sepsis as per clinical notes.

Antibiotic stewardship form:

All cases reviewed are entered in a structured form with constrained fields. A label is chosen (see Table 2). A time stamp for suspected sepsis events is ascribed according to the following rules: In case of presentation to the ER with suspected sepsis, the timestamp of presentation to triage is taken as t0. For putative sepsis events 24h post admission, the following procedure is used: If no other information is available, the sepsis order set prescription time stamp is used. If available, any initial NEWS ≥ 5 points is taken as a timestamp. If a note or specific trigger is documented by the clinical team, then the time stamp of that note can be used. In the absence of these elements, any clear deterioration (initial organ dysfunction) is used.

# **2. Variables used in the pipeline**

Variables are loaded from all wards in the clinical data warehouse, and consistency checks are performed as a preprocessing step before feature engineering or modelling.

The minimum and maximum values of the variables are indicated in Supplementary Table 19.

# **3. Scores calculations**

## 3.a. NEWS

The NEWS score [1, 6] consists of 7 independent components summarized in Supplementary Table 20.

NEWS computation is divided into 2 steps:

- Raw Data Extraction
- NEWS score computation

#### 3.a.1. Raw Data Extraction

##### 3.a.1.1. AVPU

Input variables:

- 449159002 | Alert voice pain unresponsive scale (assessment scale) |
- 248241002 | Glasgow coma score (observable entity) |

Two cases are considered:

- Case 1 – AVPU value is available
- Case 2 – AVPU value is not available, but Glasgow is.
   The AVPU value is inferred from the Glasgow coma score (GCS as follows:

GCS 15 (= 10 if intubated GCS) = A

GCS < 15 (< 10 if intubated GCS) = V, P, U

The contribution to the total NEWS score is computed as tabulated in Supplementary Table 20, IE:

**AVPU value: BP NEWS component value**

A: 0 points

V/P/U: 3 points

#### 3.a.1.2. Systolic blood pressure (SBP)

Input variables:

- 271649006 | Systolic blood pressure (observable entity) |

The contribution to the total NEWS score is computed as tabulated in Supplementary Table 20, IE:

**SBP value: BP NEWS component value**

SBP ≤ 90 mmHg: 3 points

SBP: 91-100 mmHg: 2 points

SBP: 101-110 mmHg: 1 point

SBP: 111-249 mmHg: 0 point

SBP: ≥ 250 mmHg: 1 point

##### 3.a.1.3. Fraction of inspired oxygen (FiO2)

Input variables:

- 250774007 | Inspired oxygen concentration (observable entity) |
- 427081008 | Delivered oxygen flow rate (observable entity) |

Two cases are considered:

- Case 1 – FiO2 value is available
- Case 2 – FiO2 value is not available, but the flow of delivered oxygen (O2 flow) is.
   The corresponding FiO2 value is inferred from the O2 flow as described in Supplementary Table 21 inspired from reference [7]:

The contribution to the total NEWS score is computed as tabulated in Supplementary Table 20, IE:

**FiO_2_ value: FiO_2_ NEWS component value**

21% (Room air): 0 point

>21% (any O2): 3 points

##### 3.a.1.4. Respiratory rate

Input variables:

- 86290005 | Respiratory rate (observable entity) |

The contribution to the total NEWS score is computed as tabulated in Supplementary Table 20, IE:

**RR value: RR NEWS component value**

RR ≤8 bpm: 3 points

RR 9-11 bpm: 1 point

RR; 12-20bpm: 0 point

RR: 21-24bpm: 2 points

RR ≥ 25bpm: 3 points

##### 3.a.1.5. Oxygen saturation

Input variables:

- 103228002 | Hemoglobin saturation with oxygen (observable entity) |

The contribution to the total NEWS score is computed as tabulated in Supplementary Table 20, IE:

**SpO_2_ value: SpO_2_ NEWS component value**

SpO_2_ ≤91%: 3 points

SpO_2_ 92-93%: 2 points

SpO_2_ 94-95%: 1 point

SpO_2_ ≥96%: 0 point

##### 3.a.1.6. Heart rate

Input variables:

- 364075005 | Heart rate (observable entity) |

The contribution to the total NEWS score is computed as tabulated in Supplementary Table 20, IE:

**HR value: HR NEWS component value**

HR ≤40 bpm: 2 points

HR 41-50 bpm: 1 point

HR; 51-90bpm: 0 point

HR: 91-110bpm: 1 point

HR: 111-130bpm: 2 points

HR ≥ 130bpm: 3 points

##### 3.a.1.7. Body temperature

Input variables:

- 386725007 | Body temperature (observable entity) |

The contribution to the total NEWS score is computed as tabulated in Supplementary Table 20, IE:

**Temp value: TEMP NEWS component value**

T ≤ 35.0°C: 3 points

T 35.1-36.0°C: 1 point

T 36.1-38.0°C: 0 point

T 38.1-39.0°C: 1 point

T ≥ 39.1°C: 2 points

#### 3.a.2. NEWS score computation

Each parameter is considered valid at +/- 1 hour around its measurement date. Every new value triggers the computation of a new score. For all parameters, if several measurements are recorded at the same timepoint, only the one leading to the *minimal* NEWS score is kept.

All parameters that are not available at the time of the computation are considered missing and do not contribute to the total score (i.e. they are considered as within the normal range).

The final NEWS score is obtained by summing all individual component scores.

## 3.b SOFA

The SOFA score [3] is calculated for all patients who pass through a unit where the sepsis program is deployed.

SOFA computation is divided into several steps:

- Raw Data Extraction
- Raw data preprocessing
- SOFA score computation

To provide context for the calculations described thereafter, we will use 3 dataframes as input:

- Dataframe 1: contains all patient vitals

- Dataframe 2: contains all patient laboratory analyses

- Dataframe 3: contains all medications administered to patients

#### 3.b.1. Raw Data Extraction

This step is divided into a series of several functions, each of which will extract or calculate the necessary data for the calculation of each component. Each function returns a dataframe with the same columns and containing only the relevant data.

##### 3.b.1.1. Glasgow extraction (input -> Dataframe 1, output -> df_glasgow)

Input variables:

- 248241002 | Glasgow coma score (observable entity) |

- 449159002 | Alert voice pain unresponsive scale (assessment scale) |

Two cases are handled:

- Case 1 – Glasgow value is available

o **Retrieval of the raw value of the score,** calculated by the software at CHUV

o **Retrieval of the raw score value for intubated patients.** Due to the absence of the verbal response, the value is then standardized as follows: value / 10 * 15

- Case 2 – Glasgow value is not available, but AVPU is.

o The Glasgow value is computed as follow:

**AVPU:**  **Score**

**A** **15**

**V** **12**

**P** **5**

**U** **3**

The Glasgow score is calculated whenever the data availability permits. If certain timepoints are duplicated, the median value of the scores is computed.

##### 3.b.1.2. MAP extraction (input -> Dataframe 1, output -> df_tam)

Input variables:

- 6797001 | Mean blood pressure (observable entity) |

- 271649006 | Systolic blood pressure (observable entity) |

- 271650006 | Diastolic blood pressure (observable entity) |

To extract or compute the mean arterial pressure, we extract the 3 blood pressure values: mean, systolic, and diastolic.

- Case 1 – MAP value is available

o The value is directly used. Please note that if two raw values are available at the same timepoint, the median is computed.

- Case 2 – MAP value is not available, but SAP and DAP are.

o MAP value is computed as follow: (2*SAP + DAP)/3.

If for the same timepoint, both the raw value and the calculated value are available, priority will be given to the raw value.

##### 3.b.1.3. PaO2/FiO2 computation (input -> Dataframe 1 & 2, output -> df_ratio)

Input variables:

- 103228002 | Hemoglobin saturation with oxygen (observable entity) |[DJ1] [DJ2]

- 250774007 | Inspired oxygen concentration (observable entity) |

- 25579001 | Oxygen measurement, partial pressure, arterial (procedure) |[DJ3]

- 427081008 | Delivered oxygen flow rate (observable entity) |

This step is divided into 3 sub-steps:

###### 3.b.1.3.1. PaO2 Extraction

- Case 1 – PaO2 available

The value is stored in a column named "**pao2_raw**". Please note that only arterial blood gases are used, venous gases are not considered.

- Case 2 – PaO2 is not available but O2 saturation is

PaO2 is computed as described in Supplementary Table 22.

The computed value is stored in a column named "**pao2_computed**"

###### 3.b.1.3.2. FiO2 extraction

- Case 1 – FiO2 is available

The value is stored in a column named "**fio2_raw**"

- Cas 2 – FiO2 is not available but O2 flow is.

FiO2 is computed as described in Supplementary Table 21.

FiO2 computed value is stored in a column named "**fio2_computed**"

###### 3.b.1.3.3. PaO2/ FiO2 ratio computation

For the 4 generated columns (pao2_raw, pao2_computed, fio2_raw, and fio2_computed), the data is **forward filled over 1-hour windows**. Then, a column is created for each variable (PaO2 and FiO2).

The paO2 column is generated by prioritizing pao2_raw if available, otherwise pao2_computed. The fiO2 column is generated by prioritizing fio2_raw if available, otherwise fio2_computed.

Finally, the ratio is computed using the following formula: paO2/fiO2*100. **A boolean column named "ventilated" is generated**, filled with True if the fiO2 is greater than 21, otherwise False.

##### 3.b.1.4. Labs measures extraction (input -> Dataframe 2, output -> df_labs)

Input variables (LOINC codes):

1975-2 : Bilirubin

26515-7: Platelets

2160-0: Creatinine

The values of **platelets**, **bilirubin**, and **creatinine** are extracted if available. If two values are present for the same timepoint, the median value is chosen.

##### 3.b.1.5. Medication extraction (input -> Dataframe 3, output -> df_med)

The medications are filtered by selecting only the codes corresponding to **catecholamines**:

- C01CA03 # Norepinephrine

- C01CA04 # Dopamine

- C01CA07 # Dobutamine

- C01CA24 # Epinephrine

These four medications are considered only if administered **intravenously**. An injection code is available in the CHUV data. The start and end dates of administration are also extracted. If the end administration timepoint is not indicated, a timepoint 4 hours after the injection is generated.

**Important:** The resulting dataframe will be reused in the calculation of the vasopressor indicator.

##### 3.b.1.6. Calculation of the vasopressor indicator (input -> Dataframe 1, df_med)

This function calculates an indicator for all available patient vital signs, indicating whether the patient was under vasopressor medication or not.

For all stays identified in the dataframe “**df_med**”, we extract all vital signs and join them with medication data. Then, we check for each timepoint whether the patient was under vasopressor medication or not (True or False).

##### 3.b.1.7. Extraction of urination (input -> Dataframe 1, output -> df_miction)

This function extracts the raw value of each urination and calculates the median value if two timepoints have a recorded value.

Once all the raw and/or calculated raw data is extracted, the dataframes are concatenated, except for the vasopressor indicator, which will be merged to obtain an additional “vasopressor” column with it. Missing values in this column will be filled with 0s.

#### 3.b.2. Raw data preprocessing

In our implementation, a pivot step is necessary to obtain a column for each variable. However, depending on how the data from the previous steps are aggregated, it may not be necessary.

For each of the calculated variables, **we apply a rolling window to get the last valid value within a specific time window**. Here is the list of temporal windows for each variable:

- pao2_fio2_ratio: 12H
- ventilated: 12H
- PLAQ: 24H
- Glasgow: 12H
- BILT: 72H
- TAM: 12H
- vasopressor: 12H
- CRT: 72H

#### 3.b.3. SOFA score computation

For several components, we will use the same logic: We determine in which interval the value lies. We then return the corresponding value for that interval, using a list of predefined values. For example, for the input x = 150 with intervals (99, 199, 299, 399) and values (4, 3, 2, 1, 0), the function will return the value 3.

##### 3.b.3.1. Respiratory component

The score is calculated based on whether the patient is intubated or not, according to the logic described above.

If the patient is intubated (I):

**pao2_fio2_ratio (mmHg) I** **Score**

PaO_2_ ≤ 99 4 points

PaO_2_ ≤ 199 3 points

PaO_2_ ≤ 299 2 points

PaO_2_ ≤ 399 1 point

PaO_2_ > 399 0 point

If the patient is not intubated:

**pao2_fio2_ratio (mmHg) - NI** **Score**

PaO_2_ ≤ 299 2 points

PaO_2_ ≤ 399 1 point

PaO_2_ > 399 0 point

Finally, to compute the final value of the respiratory component, for each timepoint, we check whether the patient is intubated or not to choose the value to retain.

##### 3.b.3.2. Coagulation component

According to the platelets value:

**Platelets (×10³/µL)** **Score**

Thrombocytes ≤ 19 4 points

Thrombocytes ≤ 49 3 points

Thrombocytes ≤ 99 2 points

Thrombocytes ≤ 149 1 point

Thrombocytes > 149 0 point

##### 3.b.3.3. Nervous system component

According to Glasgow coma score (GCS) :

Glasgow Score

GCS ≤ 5 4 points

GCS ≤ 9 3 points

GCS ≤ 12 2 points

GCS ≤ 14 1 point

GCS > 14 0 point

3.b.3.4. Liver component

According to bilirubin value

Bilirubin (μmol/L) score

Bilirubin > 204 4 points

Bilirubin 102-203 3 points

Bilirubin 32-101 2 points

Bilirubin 20-32 1 points

Bilirubin 0-19 0 points

##### 3.b.3.5. Cardio component

According to the value of the mean arterial pressure score, for patients without vasopressors:

Mean Arterial Pressure (mmHg) score

MAP ≤ 69 0 point

MAP > 69 1 point

For patients with vasopressors, the score is directly set to 3 points.

##### 3.b.3.6. Kidney component

According to creatinine value:

Creatinine (μmol/L) score

Creatinine 0-109 0 point

Creatinine 110-170 1 point

Creatinine 171-299 2 points

Creatinine 300-440 3 points

Creatinine > 440 4 points

In cases where creatinine is not available, but urination data is:

Urination (mL/day) score

Volume ≤ 199 4 points

Volume 200- 499 3 points

Volume > 499 0 point

##### 3.b.3.7. SOFA Computation

The SOFA score is obtained by summing all the individual component scores.

# **4. Modelisation**

The training and evaluation of Heracles are automatically executed on a monthly basis using the same dataset. This dataset aggregates all available labels up to a given date, corresponding to the last day of each month.

Training uses all available patient stays labelled through ‘sepsis program’ to train each model in the ensemble method. Evaluation is conducted on the exact same dataset using a 5-fold stratified cross-validation based on patient stays, ensuring that no stay is split between the training and validation sets.

**4.a. Training**

**Submodel 1 – Random Forest**

A total of 23 variables are used to generate 240 features (see Supplementary Table 23). Each feature is computed using a 7-day rolling window from the start to the end of the patient stay. For each window, the assigned sepsis label is the maximum label overlapping with the window. Despite the use of the 7-day rolling window, missing values may still be present. These values are imputed using the mean of all patients. The data is then scaled to unit variance. A Random Forest model is trained using the scikit-learn implementation with the following parameters:

- **n_estimators**: 200
- **max_depth**: 3
- **min_samples_split**: 2
- **min_samples_leaf**: 1
- **min_weight_fraction_leaf**: 0
- **max_features**: sqrt
- **max_leaf_nodes**: None
- **min_impurity_decrease**: 0
- **bootstrap**: True
- **oob_score**: False
- **random_state**: 42
- **warm_start**: False
- **class_weight**: None
- **ccp_alpha**: 0.0
- **max_samples**: None

**Submodel 2 – LSTM**

A total of 33 variables are used to generate 34 features (Supplementary Table 24). ‘*receive_chemotherapy*’ and *‘receive_chemotherapy_last_7days’*, are computed using ATC codes. The feature *‘receive_chemotherapy’* is derived by detecting chemotherapy drug administration (using the ATC codes listed below). The feature *‘receive_chemotherapy_last_7days’* is then generated using a 7-day rolling maximum function applied to *‘receive_chemotherapy’*.

All variables are resampled every 6 hours according to the methods specified in the table below. Features are aggregated into a 7-day tensor with dimensions *[window (4) × nb_days (7), nb_features]*. A sequential neural network with an LSTM layer (64 units) is used to capture temporal patterns. A dropout layer (0.5) is applied to prevent overfitting, followed by a dense layer (32 units) for feature transformation. The final dense layer (3 units, softmax) outputs class probabilities. The following parameters are used:

- lstm_days_window: 7
- lstm_learning_rate: 0.001
- lstm_epochs: 3
- lstm_batchsize: 16
- lstm_seed: 42

ATC codes related to chemotherapy drugs:

- L01BC07
- L01BB02
- L01XX27
- L01XX02
- L04AA04
- L01AA09
- L01FX07
- L01AB01
- L01AD01
- L01BB04
- L04AA40
- L01BB06
- L01AA01
- L01BC01
- L01XY01
- L01DB02
- L01BC08
- L01DB01
- L01CB01
- L01BB05
- L01FX02
- L01DB06
- L01FB01
- L01AA06
- B03XA06
- L01AA03
- L01BA01
- L04AX03
- L01DB07
- L01BB07
- L01XA03
- L01XX24
- L01FX14
- L01FA01
- L01AC01
- L01XX52
- L01CA02
- L01CA03

**Final Model – Random Forest**

The final model uses the probability outputs from each sub-model for each class label. These probabilities are then input into a Random Forest model, which generates the final sepsis probabilities. The model is trained using the following parameters:

- n_estimators: 200
- max_depth: 3
- min_samples_split: 5
- min_samples_leaf: 1
- min_weight_fraction_leaf: 0
- max_features: sqrt
- max_leaf_nodes: None
- min_impurity_decrease: 0
- bootstrap: False
- oob_score: False
- random_state: 42
- warm_start: False
- class_weight: None
- ccp_alpha: 0.0
- max_samples: None

**4.b Evaluation**

Evaluation is conducted using 5-fold cross-validation. For each fold, performance metrics and optimal thresholds are computed. The optimal threshold is determined to maximize precision while ensuring a minimum acceptable recall of 0.7 for the final model predicting Label 2. The final optimized threshold is computed as the mean of the optimized thresholds from each validation set.

Precision, recall, and F1-score are computed using the highest Label 2 probability per stay. The optimized threshold is then applied to determine the final stay label, which is compared to the ground truth for evaluation.

**4.c** **Indicators**

Mortality is assessed using two different methods: in-hospital mortality and 90-day mortality. In-hospital mortality is determined by checking whether the recorded date of death falls within the hospital stay, allowing for a 2-hour margin after discharge to account for data discrepancies. The 90-day mortality is calculated based on the HERACLES flag, which marks the reference point for the 90-day follow-up period.

# 5. Dashboards

See Sepsis dashboard demo.mp4

**6. References**:

1. Prytherch, David R., et al. "ViEWS—towards a national early warning score for detecting adult inpatient deterioration." *Resuscitation* 81.8 (2010): 932-937.
2. Durr, Dimitri, et al. "National Early Warning Score (NEWS) outperforms quick Sepsis-related Organ Failure (qSOFA) score for early detection of sepsis in the emergency department." *Antibiotics* 11.11 (2022): 1518.
3. Vincent, Jean-Louis, et al. "Use of the SOFA score to assess the incidence of organ dysfunction/failure in intensive care units: results of a multicenter, prospective study." *Critical care medicine* 26.11 (1998): 1793-1800.
4. Seymour, Christopher W., et al. "Assessment of clinical criteria for sepsis: for the third international consensus definitions for sepsis and septic shock (Sepsis-3)." *Jama* 315.8 (2016): 762-774.
5. Evans, Laura, et al. "Surviving sepsis campaign: international guidelines for management of sepsis and septic shock 2021." *Critical care medicine* 49.11 (2021): e1063-e1143.
6. Health Service Executive. (n.d.). The Irish National Early Warning System (INEWS) V2 User's Guide incorporating COMPASS III. Retrieved from<https://www.hse.ie/eng/about/who/cspd/ncps/deteriorating-patient-improvement-programme/inews-education-compress-training-manual.pdf>
7. European Prevalence of Infection in Intensive Care (EPIC II) study. Retrieved from<https://www.isicem.org/epic2/Documents/Estimation%20of%20PO2%20and%20FiO2.pdf>

**7.** **Supplementary Tables**

**Supplementary Table 1. Sepsis codes**

| Groups | ICD codes (ICD-10-GM) | Description |
| --- | --- | --- |
| Sepsis | A021  A207  A227  A267  A327  A40X  A41X  A427  B377  G9432  R572 | Septicaemia due to unspecified Salmonella  Septicaemic plague  Anthrax septicaemia  Erysipelothrix septicaemia  Listeria sepsis  Septicaemia due to group B streptococcus  Other septicaemias  Actinomycotic septicaemia  Candida septicaemia  Septic encephalopathy  Septic shock |

**Supplementary Table 2. CHUV Patient stay statistics**

| year | total stays | total inpatients >= 18 y.o. | Average number of stays | Number of stays with a covid19 icd code | Average admission age | Male proportion |
| --- | --- | --- | --- | --- | --- | --- |
| 2020 | 37496 | 26176 | 1.43 | 2287 | 63.37 | 52.19 |
| 2021 | 39484 | 27288 | 1.45 | 1906 | 62.89 | 52.24 |
| 2022 | 40615 | 28055 | 1.45 | 3055 | 63.26 | 50.28 |
| 2023 | 41518 | 28983 | 1.43 | 1377 | 63.15 | 51.75 |
| 2024 | 41513 | 28965 | 1.43 | 813 | 63.62 | 51.95 |

**Supplementary Table 3 CHUV all SLHS units patient stay statistics**

| year | total stays | total inpatients >= 18 y.o. | Average number of stays | Number of stays with a covid19 icd code | Average admission age | Male proportion |
| --- | --- | --- | --- | --- | --- | --- |
| 2020 | 18048 | 13834 | 1.30 | 1752 | 67.91 | 55.62 |
| 2021 | 185.96 | 14184 | 1.31 | 1417 | 67.98 | 54.78 |
| 2022 | 18704 | 14177 | 1.32 | 2038 | 68.68 | 52.42 |
| 2023 | 18719 | 14315 | 1.31 | 999 | 68.08 | 54.08 |
| 2024 | 18869 | 14293 | 1.32 | 621 | 68.40 | 52.34 |

**Supplementary Table 4. Heracles performance parameters**

|  | HERACLES cross validation (performance by 31st of August 2024) | HERACLES cross validation (performance by 31st of August 2024) | HERACLES cross validation (performance by 31st of August 2024) | HERACLES cross validation (performance by 31st of August 2024) | Test set 1 MED | Test set 2 ICD Codes |
| --- | --- | --- | --- | --- | --- | --- |
| Modelisation |  | | | | | |
| Services | MED  GIS  EMD  Other | MED | GIS | EMD | MED | MED  GIS  EMD  Other |
| Demographics |  | | | | | |
| Number of sepsis events | 1043 | 407 | 118 | 316 | 11 | 1345 |
| Number of stays | 961 | 371 | 106 | 289 | 119 | 1345 |
| Number of patients | 906 | 342 | 99 | 276 | 83 | 1180 |
| Male (%) | 575 (63.5%) | 217 | 59 | 185 | 49 (60.0%) | 720 (61.0%) |
| Female (%) | 331 (36.5%) | 125 | 40 | 91 | 34 (40.0%) | 460 (39.0%) |
| Mortality |  | | | | | |
| During hospitalization | 105 | 50 | 5 | 25 | 2 | 290 |
| HERACLES Performance |  | | | | | |
| Number of Labels  No sepsis  Pos. sepsis  Conf. sepsis | 230  341  472 | 84  136  122 | 36  27  36 | 28  65  183 | 61  47  11 | -  -  1345 |
| Precision  No sepsis  Pos. sepsis  Conf. sepsis | 0.65  0.43  0.76 | 0.52  0.43  0.64 | 0.53  0.27  0.72 | 0.40  0.39  0.85 | 1.00  0.55  0.60 | -  -  - |
| Recall  No sepsis  Pos. sepsis  Conf. sepsis | 0.16  0.74  0.64 | 0.28  0.63  0.51 | 0.68  0.33  0.5 | 0.10  0.72  0.70 | 0.52  0.96  0.27 | -  -  1.00 |
| F1-score  No sepsis  Pos. sepsis  Conf. sepsis | 0.26  0.54  0.69 | 0.36  0.51  0.57 | 0.59  0.27  0.55 | 0.16  0.51  0.77 | 0.69  0.70  0.37 | -  -  0.83 |
| AUROC  No sepsis vs Conf. sepsis | 0.88 | 0.85 | 0.86 | 0.88 | 0.92 | NA |
| AUROC  No sepsis vs Pos. & Conf. sepsis | 0.81 | 0.75 | 0.82 | 0.83 | 0.57 | NA |

NA: not applicable.

**Supplementary Table 5. Number of coded sepsis events**

| Year | SHLS wards extended | SHLS wards limited | Control wards |
| --- | --- | --- | --- |
| 2020 | 493/18836 (2.62%) | 175/11261 (1.55%) | 30/4692 (0.64%) |
| 2021 | 497/19554 (2.54%) | 198/12190 (1.62%) | 27/4776 (0.57%) |
| 2022 | 727/19733 (3.68%) | 367/12468 (2.94%) | 47/5092 (0.92%) |
| 2023 | 810/19744 (4.10%) | 401/12515 (3.20%) | 39/5485 (0.71%) |
| 2024 | 882/19692 (4.48%) | 455/12695 (3.58%) | 45/5806 (0.78%) |
| Logistic Regression | OR: 1.19 (CI 1.16-1.22) | OR: 1.27 (CI 1.23-1.32) | OR: 1.04 (CI 0.94-1.16) |

**Supplementary Table 6: Stays with HERACLES-predicted sepsis events at CHUV**

| Year | SHLS wards extended | SHLS wards limited | Control wards |
| --- | --- | --- | --- |
| 2020 | 1738/18836 (9.23%) | 482/11261 (4.28%) | 199/4692 (4.24%) |
| 2021 | 1925/19554 (9.84%) | 667/12190 (5.47%) | 223/4776 (4.67%) |
| 2022 | 2036/19733 (10.32%) | 758/12468 (6.08%) | 222/5092 (4.36%) |
| 2023 | 1960/19744 (9.93%) | 756/12515 (6.04%) | 237/5485 (4.32%) |
| 2024 | 1819/19692 (9.24%) | 655/12695 (5.16%) | 213/5806 (3.67%) |
| Logistic Regression | OR: 1.01 (CI: 0.99-1.02) | OR: 1.05 (CI: 1.02-1.07) | OR: 0.96 (CI: 0.92-1.00) |

**Supplementary Table 7. Stays with negative HERACLES-predicted sepsis events at CHUV**

| Year | SHLS wards extended | SHLS wards limited | Control wards |
| --- | --- | --- | --- |
| 2020 | 13415/18836 (71.22%) | 8254/11261 (73.30%) | 2804/4692 (59.76%) |
| 2021 | 13877/19554 (70.97%) | 8912/12190 (73.11%) | 2882/4776 (60.34%) |
| 2022 | 13682/19733 (69.34%) | 8770/12468 (70.34%) | 2938/5092 (57.70%) |
| 2023 | 13840/19744 (70.10%) | 8890/12515 (71.03%) | 3080/5485 (56.16%) |
| 2024 | 12594/19692 (63.95%) | 7988/12695 (62.92%) | 3115/5806 (53.64%) |
| Logistic Regression | OR: 0.92 (CI: 0.92-0.93) | OR: 0.89 (CI: 0.88-0.90) | OR: 0.93 (CI: 0.92-0.95) |

**Supplementary Table 8. In-hospital mortality in stays with HERACLES-predicted sepsis event**

| Year | SHLS wards extended | SHLS wards limited | Control wards |
| --- | --- | --- | --- |
| 2020 | 386/1738 (22.21%) | 99/482 (20.75%) | 13/199 (6.53%) |
| 2021 | 377/1925 (19.58%) | 127/667 (19.34%) | 20/223 (8.97%) |
| 2022 | 436/2036 (21.41%) | 124/758 (16.62%) | 22/222 (9.91%) |
| 2023 | 330/1960 (16.84%) | 99/756 (13.49%) | 21/237 (8.86%) |
| 2024 | 323/1819 (17.76%) | 100/655 (15.57%) | 25/213 (11.74%) |
| Logistic Regression | OR: 0.93 (CI: 0.89-0.96) | OR: 0.89 (CI: 0.83-0.96) | OR: 1.13 (CI: 0.97-1.32) |

**Supplementary Table 9. In-hospital mortality in stays with negative HERACLES-predicted sepsis event**

| Year | SHLS wards extended | SHLS wards limited | Control wards |
| --- | --- | --- | --- |
| 2020 | 163/13415(1.21%) | 71/8254(0.86%) | 12/2804 (0.43%) |
| 2021 | 174/13877(1.25%) | 88/8912(0.99%) | 6/2882 (0.21%) |
| 2022 | 176/13682(1.29%) | 98/8770(1.11%) | 22/2938 (0.75%) |
| 2023 | 148/13840(1.07%) | 67/8890(0.75%) | 16/3080 (0.52%) |
| 2024 | 178/12594(1.41%) | 73/7988(0.91%) | 13/3115 (0.42%) |
| Logistic Regression | OR: 1.01 (CI: 0.96-1.06) | OR: 0.98 (CI: 0.91-1.05) | OR: 1.02 (CI: 0.86-1.21) |

**Supplementary Table 10. 90-day mortality in stays with HERACLES-predicted sepsis event**

| Year | SHLS wards extended | SHLS wards limited | Control wards |
| --- | --- | --- | --- |
| 2020 | 517/1738 (29.75%) | 159/482 (32.99%) | 25/199 (12.56%) |
| 2021 | 522/1925 (27.12%) | 209/667 (31.33%) | 35/223 (15.70%) |
| 2022 | 616/2036 (30.26%) | 224/758 (29.55%) | 33/222 (14.86%) |
| 2023 | 479/1960 (24.44%) | 184/756 (24.34%) | 34/237 (14.35%) |
| 2024 | 455/1819 (25.01%) | 171/655 (26.11%) | 32/213 (15.02%) |
| Logistic Regression | OR: 0.94 (CI: 0.91-0.97) | OR: 0.91 (CI: 0.86-0.97) | OR: 1.04 (CI: 0.92-1.19) |

**Supplementary Table 11. 90-day mortality in stays with negative HERACLES-predicted sepsis event**

| Year | SHLS wards extended | SHLS wards limited | Control wards |
| --- | --- | --- | --- |
| 2020 | 866/13415(6.46%) | 508/8254(6.15%) | 114/2804 (4.07%) |
| 2021 | 913/13877(6.58%) | 596/8912(6.69%) | 100/2882 (3.47%) |
| 2022 | 848/13682(6.20%) | 576/8770(6.57%) | 104/2938 (3.54%) |
| 2023 | 755/13840(5.46%) | 483/8890(5.43%) | 104/3080 (3.38%) |
| 2024 | 798/12594(6.34%) | 512/7988(6.41%) | 99/3115 (3.18%) |
| Logistic Regression | OR: 0.97 (CI: 0.95-0.99) | OR: 0.98 (CI: 0.95-1.01) | OR: 0.91 (CI: 0.86-0.97) |

**Supplementary Table 12. Logistic Regression values**

| Entity | GROUP | outcome | variable | p_value | z_score | coef | stderr | OR | Lower CI | Upper CI |
| --- | --- | --- | --- | --- | --- | --- | --- | --- | --- | --- |
| Sepsis code | Incl. SLHS wards |  | year | 0 | 0 | 0.1753 | 0.0129 | 1.1916 | 1.1619 | 1.222 |
| Sepsis code | Incl. SLHS wards |  | age | 0 | 0 | 0.0125 | 0.0011 | 1.0126 | 1.0104 | 1.0147 |
| Sepsis code | Incl. SLHS wards |  | sex | 0 | 0 | -0.4467 | 0.0372 | 0.6398 | 0.5947 | 0.6882 |
| Sepsis code | Incl. SLHS wards |  | CCI | 0 | 0 | 0.2215 | 0.005 | 1.2479 | 1.2357 | 1.2602 |
| Sepsis code | Lim. SLHS wards |  | year | 0 | 0 | 0.2411 | 0.0192 | 1.2726 | 1.2257 | 1.3213 |
| Sepsis code | Lim. SLHS wards |  | age | 0 | 0 | 0.0269 | 0.0017 | 1.0273 | 1.024 | 1.0306 |
| Sepsis code | Lim. SLHS wards |  | sex | 0 | 0 | -0.5244 | 0.0536 | 0.5919 | 0.5329 | 0.6574 |
| Sepsis code | Lim. SLHS wards |  | CCI | 0 | 0 | 0.1729 | 0.0078 | 1.1888 | 1.1707 | 1.2071 |
| Sepsis code | Ctrl wards |  | year | 0.3859 | 0.3859 | 0.0455 | 0.0525 | 1.0466 | 0.9442 | 1.16 |
| Sepsis code | Ctrl wards |  | age | 0.0004 | 0.0004 | 0.0197 | 0.0056 | 1.0199 | 1.0089 | 1.0311 |
| Sepsis code | Ctrl wards |  | sex | 0 | 0 | -0.78 | 0.1768 | 0.4584 | 0.3242 | 0.6482 |
| Sepsis code | Ctrl wards |  | CCI | 0 | 0 | 0.2869 | 0.0207 | 1.3324 | 1.2794 | 1.3875 |
|  |  |  |  |  |  |  |  |  |  |  |
| HERACLES stay | Incl. SLHS wards |  | year | 0.2783 | 0.2783 | 0.0085 | 0.0079 | 1.0086 | 0.9931 | 1.0242 |
| HERACLES stay | Incl. SLHS wards |  | age | 0 | 0 | 0.0053 | 0.0006 | 1.0053 | 1.0041 | 1.0065 |
| HERACLES stay | Incl. SLHS wards |  | sex | 0 | 0 | -0.4286 | 0.0229 | 0.6514 | 0.6229 | 0.6813 |
| HERACLES stay | Incl. SLHS wards |  | CCI | 0 | 0 | 0.1815 | 0.0036 | 1.1991 | 1.1907 | 1.2074 |
| HERACLES stay | Lim. SLHS wards |  | year | 0.0002 | 0.0002 | 0.0488 | 0.013 | 1.05 | 1.0235 | 1.0772 |
| HERACLES stay | Lim. SLHS wards |  | age | 0 | 0 | 0.0181 | 0.0011 | 1.0183 | 1.0161 | 1.0204 |
| HERACLES stay | Lim. SLHS wards |  | sex | 0 | 0 | -0.3856 | 0.0372 | 0.68 | 0.6322 | 0.7315 |
| HERACLES stay | Lim. SLHS wards |  | CCI | 0 | 0 | 0.1672 | 0.0058 | 1.182 | 1.1688 | 1.1954 |
| HERACLES stay | Ctrl wards |  | year | 0.0707 | 0.0707 | -0.0395 | 0.0219 | 0.9613 | 0.9209 | 1.0033 |
| HERACLES stay | Ctrl wards |  | age | 0 | 0 | 0.0116 | 0.0021 | 1.0116 | 1.0074 | 1.0159 |
| HERACLES stay | Ctrl wards |  | sex | 0 | 0 | -0.5648 | 0.0694 | 0.5685 | 0.4962 | 0.6513 |
| HERACLES stay | Ctrl wards |  | CCI | 0 | 0 | 0.1142 | 0.0111 | 1.1209 | 1.0969 | 1.1455 |
|  |  |  |  |  |  |  |  |  |  |  |
| HERACLES stay | Incl. SLHS wards | In-hosp. mort. | year | 0.0001 | 0.0001 | -0.074 | 0.019 | 0.9286 | 0.8946 | 0.9639 |
| HERACLES stay | Incl. SLHS wards | In-hosp. mort. | age | 0 | 0 | 0.0286 | 0.0019 | 1.029 | 1.0252 | 1.0329 |
| HERACLES stay | Incl. SLHS wards | In-hosp. mort. | sex | 0.3766 | 0.3766 | -0.0487 | 0.0551 | 0.9525 | 0.855 | 1.061 |
| HERACLES stay | Incl. SLHS wards | In-hosp. mort. | CCI | 0 | 0 | 0.0475 | 0.008 | 1.0486 | 1.0324 | 1.0651 |
| HERACLES stay | Lim. SLHS wards | In-hosp. mort. | year | 0.0019 | 0.0019 | -0.112 | 0.0361 | 0.8941 | 0.833 | 0.9596 |
| HERACLES stay | Lim. SLHS wards | In-hosp. mort. | age | 0 | 0 | 0.0527 | 0.0043 | 1.0541 | 1.0452 | 1.0631 |
| HERACLES stay | Lim. SLHS wards | In-hosp. mort. | sex | 0.8508 | 0.8508 | 0.0186 | 0.0989 | 1.0188 | 0.8393 | 1.2367 |
| HERACLES stay | Lim. SLHS wards | In-hosp. mort. | CCI | 0 | 0 | 0.0755 | 0.0152 | 1.0785 | 1.0468 | 1.1112 |
| HERACLES stay | Ctrl wards | In-hosp. mort. | year | 0.1209 | 0.1209 | 0.1223 | 0.0789 | 1.1301 | 0.9683 | 1.319 |
| HERACLES stay | Ctrl wards | In-hosp. mort. | age | 0 | 0 | 0.0402 | 0.0096 | 1.041 | 1.0216 | 1.0608 |
| HERACLES stay | Ctrl wards | In-hosp. mort. | sex | 0.2729 | 0.2729 | 0.2555 | 0.233 | 1.2911 | 0.8177 | 2.0384 |
| HERACLES stay | Ctrl wards | In-hosp. mort. | CCI | 0 | 0 | 0.2006 | 0.0324 | 1.2222 | 1.1469 | 1.3024 |
|  |  |  |  |  |  |  |  |  |  |  |
| HERACLES stay | Incl. SLHS wards | D-90 mort. | year | 0.0004 | 0.0004 | -0.0615 | 0.0173 | 0.9404 | 0.9091 | 0.9728 |
| HERACLES stay | Incl. SLHS wards | D-90 mort. | age | 0 | 0 | 0.0366 | 0.0018 | 1.0373 | 1.0337 | 1.0409 |
| HERACLES stay | Incl. SLHS wards | D-90 mort. | sex | 0.1238 | 0.1238 | -0.077 | 0.05 | 0.9259 | 0.8394 | 1.0213 |
| HERACLES stay | Incl. SLHS wards | D-90 mort. | CCI | 0 | 0 | 0.0886 | 0.0072 | 1.0926 | 1.0772 | 1.1082 |
| HERACLES stay | Lim. SLHS wards | D-90 mort. | year | 0.0017 | 0.0017 | -0.0948 | 0.0303 | 0.9096 | 0.8572 | 0.9651 |
| HERACLES stay | Lim. SLHS wards | D-90 mort. | age | 0 | 0 | 0.0468 | 0.0034 | 1.0479 | 1.041 | 1.0549 |
| HERACLES stay | Lim. SLHS wards | D-90 mort. | sex | 0.5899 | 0.5899 | -0.0448 | 0.0832 | 0.9562 | 0.8123 | 1.1255 |
| HERACLES stay | Lim. SLHS wards | D-90 mort. | CCI | 0 | 0 | 0.1286 | 0.013 | 1.1372 | 1.1085 | 1.1667 |
| HERACLES stay | Ctrl wards | D-90 mort. | year | 0.513 | 0.513 | 0.0438 | 0.067 | 1.0448 | 0.9162 | 1.1915 |
| HERACLES stay | Ctrl wards | D-90 mort. | age | 0 | 0 | 0.057 | 0.0086 | 1.0587 | 1.0409 | 1.0767 |
| HERACLES stay | Ctrl wards | D-90 mort. | sex | 0.3866 | 0.3866 | 0.1758 | 0.203 | 1.1921 | 0.8008 | 1.7747 |
| HERACLES stay | Ctrl wards | D-90 mort. | CCI | 0 | 0 | 0.2502 | 0.0296 | 1.2843 | 1.2118 | 1.3611 |

Abbreviations: CCI – Charlson’s Comorbidity Index, Ctrl – control, Lim. – limited, Incl. – including, mort. – mortality, “Stays including SLHS wards” which consist of patient stay during which the patient spent any portion of their stay in at least one ward where the SLHS was deployed. “Stays limited to SLHS wards” which consist of patient stays confined entirely to wards where the SLHS was deployed.

**Supplementary Table 13. Time to antibiotics for confirmed sepsis – cohort description**

|  | t ≤ 1 hour | 1 hour < t ≤ 3 hours | t > 3 hours |
| --- | --- | --- | --- |
|  | (without / with pathway) | (without / with pathway) | (without / with pathway) |
| Documented cases | 25 / 56 | 52 / 44 | 150 / 121 |
| Age at sepsis | 60.0 (39.4, 84.8) / 65.5 (45.0, 81.5) | 65.0 (34.3, 86.7) / 68.5 (39.9, 84.1) | 68.0 (51.0, 87.0) / 70.0 (51.0, 88.0) |
| Charlson Comorbidity Index | 2.0 (0.0, 8.0) / 3.0 (0.0, 8.0) | 2.5 (0.0, 9.0) / 6.0 (0.0, 8.0) | 3.0 (0.0, 9.0) / 4.0 (1.0, 9.0) |
| Lactate Max | 2.46 (1.5, 8.6) / 2.46 (1.3, 7.8) | 2.84 (1.6, 6.9) / 3.0 (1.5, 5.8) | 2.39 (1.2, 5.7) / 2.51 (1.1, 6.0) |
| LOS After Sepsis (days) | 13.0 (4.0, 23.2) / 15.0 (5.0, 25.5) | 11.0 (3.0, 44.6) / 12.0 (4.0, 30.1) | 12.0 (4.0, 48.1) / 11.0 (4.0, 39.0) |
| Time to Antibiotics (hours) | 0.55 (0.2, 0.8) / 0.62 (0.0, 1.0) | 1.97 (1.3, 2.7) / 1.99 (1.2, 2.8) | 6.48 (3.5, 21.9) / 5.5 (3.7, 17.0) |
| Time to ICU (hours) | 4.48 (2.1, 28.0) / 4.8 (2.1, 10.9) | 9.83 (2.8, 40.4) / 5.57 (2.6, 16.2) | 12.08 (1.7, 33.2) / 8.98 (2.3, 32.8) |
| Death in 30 Days | 4 (16.0%) / 10 (17.9%) | 11 (21.2%) / 4 (9.1%) | 21 (14.0%) / 19 (15.7%) |
| Death in 90 Days | 7 (28.0%) / 11 (19.6%) | 13 (25.0%) / 11 (25.0%) | 31 (20.7%) / 26 (21.5%) |
| Sex masculine | 15 (60.0%) / 40 (71.4%) | 31 (59.6%) / 32 (72.7%) | 98 (65.3%) / 94 (77.7%) |

**Supplementary Table 14. Time to antibiotics for confirmed sepsis – contingency tables corrected for age, sex, and Charlson**

|  | t ≤ 1 hour | t > 1 hour |
| --- | --- | --- |
| Without Pathway | 19 | 202 |
| With Pathway | 56 | 165 |
| χ2 = 20.81, p < 0.001 |  |  |

**Supplementary Table 15. Time to antibiotics for confirmed sepsis – contingency tables corrected for age, sex, and Charlson**

|  | t ≤ 3 hours | t > 3 hours |
| --- | --- | --- |
| Without Pathway | 78 | 143 |
| With Pathway | 100 | 121 |
| χ2 = 4.15, p = 0.042 |  |  |

**Supplementary Table 16. Time to antibiotics for confirmed and possible sepsis – cohort description**

|  | t ≤ 1 hour | 1 hour < t ≤ 3 hours | t > 3 hours |
| --- | --- | --- | --- |
|  | (without / with pathway) | (without / with pathway) | (without / with pathway) |
| Documented cases | 38 / 111 | 75 / 81 | 222 / 240 |
| Age at sepsis | 60.0 (39.7, 82.3) / 65.0 (46.0, 85.0) | 66.0 (34.8, 83.6) / 66.0 (38.0, 82.0) | 69.0 (51.0, 87.0) / 69.0 (43.0, 87.0) |
| Charlson Comorbidity Index | 3.0 (0.0, 8.0) / 3.0 (0.0, 10.0) | 3.0 (0.0, 9.0) / 6.0 (0.0, 9.0) | 3.0 (0.0, 8.0) / 3.0 (0.0, 9.0) |
| Lactate Max | 2.46 (1.4, 8.2) / 2.21 (1.1, 5.9) | 2.72 (1.5, 6.9) / 2.21 (1.3, 5.0) | 2.37 (1.2, 5.6) / 1.92 (1.1, 5.2) |
| LOS After Sepsis (days) | 15.0 (4.0, 28.3) / 12.0 (5.0, 32.0) | 11.0 (3.0, 48.0) / 11.0 (4.0, 36.0) | 11.0 (4.0, 40.9) / 11.0 (4.0, 34.1) |
| Time to Antibiotics (hours) | 0.53 (0.1, 0.9) / 0.5 (0.1, 1.0) | 2.05 (1.2, 2.7) / 1.78 (1.2, 2.8) | 6.81 (3.6, 21.8) / 6.08 (4.0, 22.1) |
| Time to ICU (hours) | 4.9 (1.8, 64.9) / 5.81 (2.0, 137.3) | 10.34 (3.4, 76.2) / 6.38 (2.7, 50.6) | 13.21 (2.2, 45.7) / 8.57 (2.5, 54.3) |
| Death in 30 Days | 8 (21.1%) / 17 (15.3%) | 13 (17.3%) / 10 (12.3%) | 29 (13.1%) / 34 (14.2%) |
| Death in 90 Days | 11 (28.9%) / 22 (19.8%) | 16 (21.3%) / 20 (24.7%) | 47 (21.2%) / 51 (21.2%) |
| Sex masculine | 22 (57.9%) / 70 (63.1%) | 44 (58.7%) / 56 (69.1%) | 148 (66.7%) / 170 (70.8%) |

**Supplementary Table 17. Time to antibiotics for confirmed and possible sepsis – contingency table corrected for age, sex, and Charlson**

|  | t ≤ 1 hour | t > 1 hour |
| --- | --- | --- |
| Without Pathway | 58 | 374 |
| With Pathway | 111 | 321 |
| χ2 = 19.89, p < 0.001 |  |  |

**Supplementary Table 18. Time to antibiotics for confirmed and possible sepsis – contingency table corrected for age, sex, and Charlson**

|  | t ≤ 3 hours | t > 3 hours |
| --- | --- | --- |
| Without Pathway | 151 | 281 |
| With Pathway | 192 | 240 |
| χ2 = 7.74, p = 0.054 |  |  |

**Supplementary Table 19. Minimum and maximum values of the variables of the pipeline**

|  | metadata | variable_name | Min value | Max value | Unit | Standard | Code/Name |
| --- | --- | --- | --- | --- | --- | --- | --- |
| Patient |  | patient_id |  |  |  | LOINC | 76435-7 |
|  |  | stay_id |  |  |  | LOINC | 94659-0 |
|  |  | stay_start_date |  |  |  | SNOMED | 399423000 |
|  |  | stay_end_date |  |  |  | SNOMED | 442864001 |
|  |  | unit_start_date |  |  |  |  |  |
|  |  | unit_end_date |  |  |  |  |  |
|  |  | unit_name |  |  |  |  |  |
|  |  | unit_id |  |  |  | SNOMED | 568291000005106 |
|  |  | department_id |  |  |  | SNOMED | 284548004 |
|  |  | department_name |  |  |  |  |  |
| demographics |  | date_of_birth |  |  |  | SNOMED | 184099003 |
|  |  | date_of_death |  |  |  | SNOMED | 399753006 |
|  |  | sex |  |  |  | SNOMED | 184100006 |
| Sepsis_event |  | t0_sepsis |  |  |  |  |  |
|  |  | sepsis_id |  |  |  |  |  |
|  |  | clinician_reviewer |  |  |  |  |  |
|  |  | label |  |  |  |  |  |
|  |  | label_text |  |  |  |  |  |
| Vital sign | Time | weight | 10 | 260 | kg | SNOMED | 27113001 |
|  | value | FiO2 | 1 | 100 | % | SNOMED | 250774007 |
|  |  | Heart rate | 20 | 240 | bpm | SNOMED | 364075005 |
|  |  | Respiratory rate | 1 | 160 | bpm | SNOMED | 86290005 |
|  |  | o2_saturation | 1 | 100 | % | SNOMED | 103228002 |
|  |  | temperature | 27 | 45 | Celsius | SNOMED | 386725007 |
|  |  | systolic_blood_pressure | 0 | 250 | mmHg | SNOMED | 271649006 |
|  |  | diastolic_blood_pressure | 0 | 250 | mmHg | SNOMED | 271650006 |
|  |  | mean blood pressure | 0 | 250 | mmHg | SNOMED | 6797001 |
|  |  | avpu |  |  |  | SNOMED | 449159002 |
|  |  | o2_flow | 1 | 100 | l/min | SNOMED | 427081008 |
|  |  | miction / urine output | 0 | 10000 | ml | SNOMED | 364202003 |
|  |  | glasgow_score | 3 | 15 |  | SNOMED | 386554004 |
| Labs | date_sample | bilirubin | 0 | 1100 | µmol/l | LOINC | 14631-6 |
|  | loinc_code | creatinine | 0 | 4000 | µmol/l | LOINC | 14682-9 |
|  | chuv_code | lactate | 0 | 10 | mmol/l | LOINC | 32693-4 |
|  | value | platelets | 0 | 5000 | g/l | LOINC | 777-3 |
|  |  | c_reactive_protein | 0 | 1000 | mg/l | LOINC | 1988-5 |
|  |  | pao2 | 0 | 200 | mmHg | LOINC | 71841-1 |
|  |  | procalcitonin | 0 | 2000 | µg/l | LOINC | 33959-8 |
| Medication | atc_code | All |  |  |  |  |  |
|  | atc_name |  |  |  |  |  |  |
|  | date_start_admin |  |  |  |  |  |  |
|  | date_end_admin |  |  |  |  |  |  |
|  | administration |  |  |  |  |  |  |

**Supplementary Table 20. National early warning score**

| National Early Warning Score | | | | | | | |
| --- | --- | --- | --- | --- | --- | --- | --- |
| Score | 3 | 2 | 1 | 0 | 1 | 2 | 3 |
| Respiratory rate (bpm) | ≤8 |  | 9-11 | 12-20 |  | 21-24 | ≥25 |
| SpO2 (%) | ≤91 | 92-93 | 94-95 | ≥96 |  |  |  |
| Inspired O2 (FiO2) |  |  |  | Air |  |  | Any O2 |
| Heart Rate (bpm) |  | ≤40 | 41-50 | 51-90 | 91-110 | 111-130 | ≥131 |
| Systolic BP (mmHg) | ≤90 | 91-100 | 101-110 | 111-249 | ≥250 |  |  |
| AVCPU |  |  |  | Alert (A) |  |  | Voice (V), pain (P), unresponsive (U) |
| Temp (C°) | ≤35.0 |  | 35.1-36.0 | 36.1-38.0 | 38.1-39.0 | ≥39.1 |  |

**Supplementary Table 21. O_2_ to FiO_2_ conversion rate**

| **O2 Flow (l/min)** | **FiO2 (%)** |
| --- | --- |
| 0 | 21 |
| 1 | 24 |
| 2 | 28 |
| 3 | 32 |
| 4 | 36 |
| 5 | 40 |
| 6 | 50 |
| 7 | 60 |
| 8 | 60 |
| 9 | 90 |
| 10 | 95 |

**Supplementary Table 22. O_2_ to PaO_2_ conversion rate**

| **O2 saturation (%)** | **PaO2 (mmHg)** |
| --- | --- |
| <80 | 44 |
| 80 | 44 |
| 81 | 45 |
| 82 | 46 |
| 83 | 47 |
| 84 | 49 |
| 85 | 50 |
| 86 | 52 |
| 87 | 53 |
| 88 | 55 |
| 89 | 57 |
| 90 | 60 |
| 91 | 62 |
| 92 | 65 |
| 93 | 69 |
| 94 | 73 |
| 95 | 79 |
| 96 | 86 |
| 97 | 96 |
| 98 | 112 |
| 99 | 145 |

**Supplementary Table 23. Random Forest variables**

| Sub model 1 - Random Forest - Variables and Features | | |
| --- | --- | --- |
| Variables | Features | Description |
| AVPU | avpu_a | Compute dummies feature using AVPU value |
|  | avpu_v |  |
|  | avpu_p |  |
|  | avpu_u |  |
| bilirubin | bilirubin_std | compute standard deviation |
|  | bilirubin_min | compute minimum value |
|  | bilirubin_max | compute maximum value |
|  | bilirubin_mean | compute mean value |
|  | bilirubin_nb | count how many times bilirubin has been measured |
|  | bilirubin_nb_higher | count values higher than 21 |
|  | bilirubin_is_measured | Boolean indicating if it has been measured |
| creatinine | creatinine_std | compute standard deviation |
|  | creatinine_min | compute minimum value |
|  | creatinine_max | compute maximum value |
|  | creatinine_mean | compute mean value |
|  | creatinine_nb | count how many times creatinine has been measured |
|  | creatinine_nb_higher | count values higher than 110 (for men) or 90 (for women) |
|  | creatinine_nb_lower | count values lower than 60 (for men) or 45 (for women) |
|  | creatinine_is_measured | Boolean indicating if it has been measured |
| CRP | crp_std | compute standard deviation |
|  | crp_min | compute minimum value |
|  | crp_max | compute maximum value |
|  | crp_mean | compute mean value |
|  | crp_nb | count how many times crp has been measured |
|  | crp_nb_higher | count values higher than 10 |
|  | crp_is_measured | Boolean indicating if it has been measured |
| diastolic | diastolic_std | compute standard deviation |
|  | diastolic_min | compute minimum value |
|  | diastolic_max | compute maximum value |
|  | diastolic_mean | compute mean value |
|  | diastolic_nb | count how many times diastolic has been measured |
|  | diastolic_nb_higher | count values higher than 80 |
|  | diastolic_nb_lower | count values lower than 60 |
|  | diastolic_is_measured | Boolean indicating if it has been measured |
| systolic | systolic_std | compute standard deviation |
|  | systolic_min | compute minimum value |
|  | systolic_max | compute maximum value |
|  | systolic_mean | compute mean value |
|  | systolic_nb | count how many times systolic has been measured |
|  | systolic_nb_higher | count values higher than 120 |
|  | systolic_nb_lower | count values lower than 90 |
|  | systolic_is_measured | Boolean indicating if it has been measured |
| mean | mean_std | compute standard deviation |
|  | mean_min | compute minimum value |
|  | mean_max | compute maximum value |
|  | mean_mean | compute mean value |
|  | mean_nb | count how many times mean has been measured |
|  | mean_nb_higher | count values higher than 100 |
|  | mean_nb_lower | count values lower than 60 |
|  | mean_is_measured | Boolean indicating if it has been measured |
| heartrate | heartrate_std | compute standard deviation |
|  | heartrate_min | compute minimum value |
|  | heartrate_max | compute maximum value |
|  | heartrate_mean | compute mean value |
|  | heartrate_nb | count how many times heartrate has been measured |
|  | heartrate_nb_higher | count values higher than 100 |
|  | heartrate_nb_lower | count values lower than 60 |
|  | heartrate_is_measured | Boolean indicating if it has been measured |
| lactate | lactate_std | compute standard deviation |
|  | lactate_min | compute minimum value |
|  | lactate_max | compute maximum value |
|  | lactate_mean | compute mean value |
|  | lactate_nb | count how many times lactate has been measured |
|  | lactate_nb_higher | count values higher than 2 |
|  | lactate_is_measured | Boolean indicating if it has been measured |
| drug_atc_code | "A01" | Compute dummies features using highest level of ATC codes (97 columns are computed) |
|  | … |  |
|  | "V11" |  |
| Oxygen inspired fraction | oxygeninspiredfraction_std | compute standard deviation |
|  | oxygeninspiredfraction_min | compute minimum value |
|  | oxygeninspiredfraction_max | compute maximum value |
|  | oxygeninspiredfraction_mean | compute mean value |
|  | oxygeninspiredfraction_nb | count how many times oxygen inspired fraction has been measured |
|  | oxygeninspiredfraction_nb_higher | count values higher than 21 |
|  | oxygeninspiredfraction_is_measured | Boolean indicating if it has been measured |
| Oxygen saturation | oxygensaturation_std | compute standard deviation |
|  | oxygensaturation_min | compute minimum value |
|  | oxygensaturation_max | compute maximum value |
|  | oxygensaturation_mean | compute mean value |
|  | oxygensaturation_nb | count how many times oxygen saturation has been measured |
|  | oxygensaturation_nb_lower | count values lower than 95 |
|  | oxygensaturation_is_measured | Boolean indicating if it has been measured |
| platelets | platelets_std | compute standard deviation |
|  | platelets_min | compute minimum value |
|  | platelets_max | compute maximum value |
|  | platelets_mean | compute mean value |
|  | platelets_nb | count how many times platelets has been measured |
|  | platelets_nb_higher | count values higher than 400 |
|  | platelets_nb_lower | count values lower than 150 |
|  | platelets_is_measured | Boolean indicating if it has been measured |
| procalcitonine | procalcitonine_std | compute standard deviation |
|  | procalcitonine_min | compute minimum value |
|  | procalcitonine_max | compute maximum value |
|  | procalcitonine_mean | compute mean value |
|  | procalcitonine_nb | count how many times procalcitonine has been measured |
|  | procalcitonine_nb_higher | count values higher than 1 |
|  | procalcitonine_is_measured | Boolean indicating if it has been measured |
| temperature | temperature_std | compute standard deviation |
|  | temperature_min | compute minimum value |
|  | temperature_max | compute maximum value |
|  | temperature_mean | compute mean value |
|  | temperature_nb | count how many times temperature has been measured |
|  | temperature_nb_higher | count values higher than 38 |
|  | temperature_is_measured | Boolean indicating if it has been measured |
| weight | weight_std | compute standard deviation |
|  | weight_min | compute minimum value |
|  | weight_max | compute maximum value |
|  | weight_mean | compute mean value |
|  | weight_nb | count how many times weight has been measured |
|  | weight_is_measured | Boolean indicating if it has been measured |
| Infection | infection | compute a dummy feature |
| SOFA | max_dsofa | compute the maximum delta sofa |
| NEWS | max_news | compute the maximum news |
| Date of birth | age |  |
| sex | sex |  |
| unit | unit_[…] | compute n dummies feature indicating in which unit the patient is currently hospitalized |

**Supplementary Table 24. Long Short-Term Memory variables**

| Sub model 2 - LSTM - Variables and Features | | |
| --- | --- | --- |
| Variable | Feature | Description |
| creatinine | sofa_algo_scorekidney | max followed by forward fill and backward fill (grouped by stay) |
| Bilirubin | sofa_algo_scoreliver |  |
| PaO2 andFiO2 | sofa_algo_scorerespiratory |  |
| Glasgow | sofa_algo_scorenervoussystem |  |
| platelets | sofa_algo_scorecoagulation |  |
| Mean arterial pressure | sofa_algo_scorecardiovasculary |  |
| sofa | sofa_score |  |
| avpu | news_algo_scoreavpu |  |
| heart rate | news_algo_scoreheartrate |  |
| oxygen inspired | news_algo_scoreoxygeninspiredrate |  |
| oxygen saturation | news_algo_scoreoxygensaturation |  |
| respiratory rate | news_algo_scorerespiratoryrate |  |
| systolic | news_algo_scoresystolicbloodpressure |  |
| temperature | news_algo_scoretemperature |  |
| news | news_algo |  |
| infection | putativesepsisevent_infection |  |
| ATC codes | receive_chimiotherapy |  |
| ATC codes | receive_chimiotherapy_last_7days |  |
| bilirubin | bilirubin | mean followed by linear interpolation then forward fill and backward fill (grouped by stay) |
| creatinine | creatinine |  |
| crp | crp |  |
| diastolic | diastolic |  |
| glasgow score | glasgow score |  |
| heart rate | heart rate |  |
| lactate | lactate |  |
| mean | mean |  |
| oxygen saturation | oxygen saturation |  |
| oxygen inspired | oxygen inspired |  |
| platelets | platelets |  |
| procalcitonin | procalcitonin |  |
| respiratory rate | respiratory rate |  |
| systolic | systolic |  |
| temperature | temperature |  |
| weight | weight |  |

**8.** **Supplementary Figures**


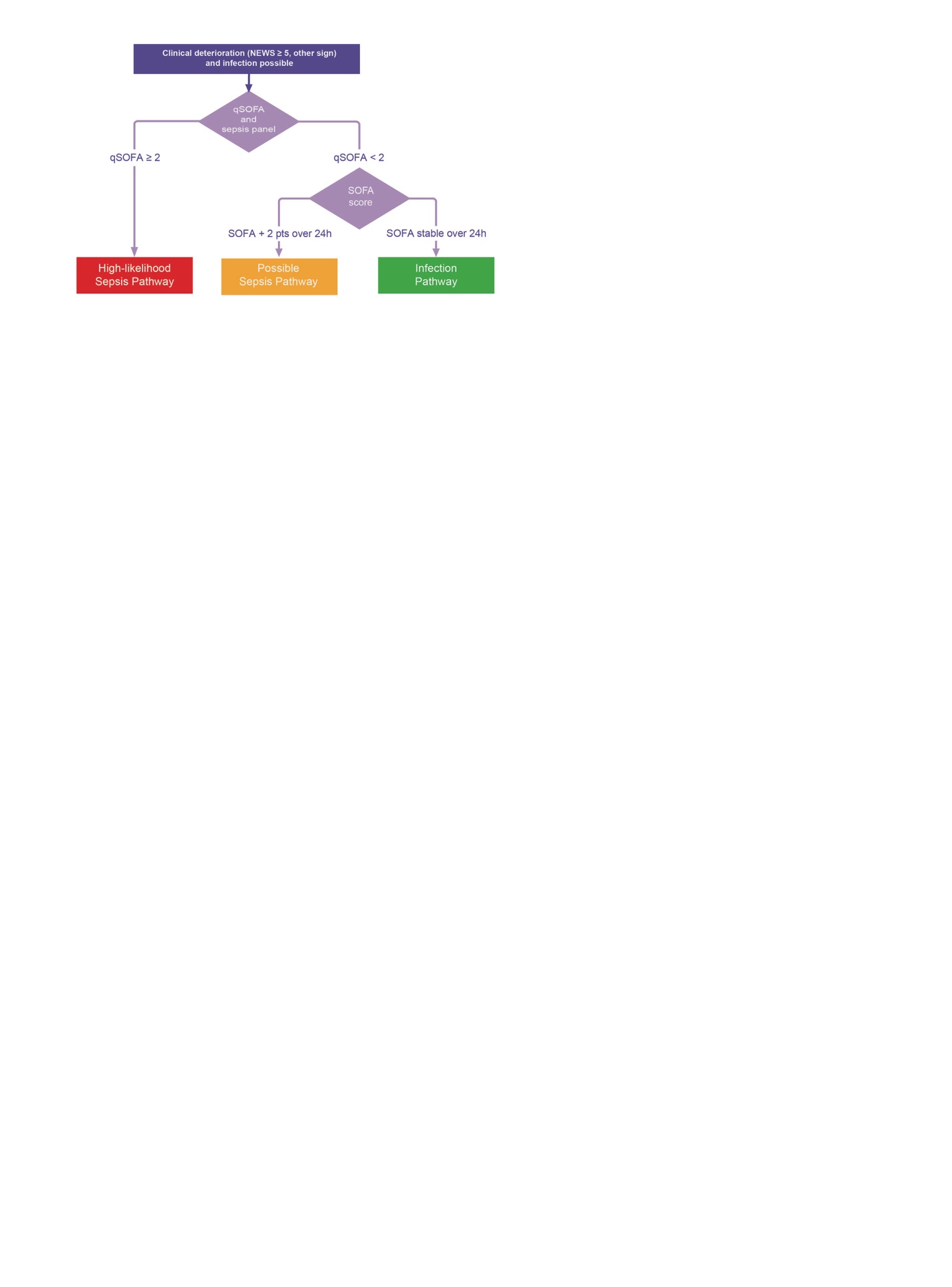


**Supplementary Figure 1. Sepsis program pathway.** Decision algorithm according to deterioration of patient to determine urgency of care. The qSOFA score is a bedside tool used to identify patients at risk of sepsis-related complications based on altered mental status, low blood pressure, and rapid respiratory rate, helping clinicians to quickly assess and manage sepsis outside the intensive care setting. High-likelihood (red) sepsis pathway calls for a management bundle (antibiotics, fluids and source control strategy) within 1 hour. Possible (orange) sepsis pathway calls for a management bundle (antibiotics, fluids and source control strategy) within 3 hours. The Infection (green) pathway calls for antibiotics as deemed appropriate.


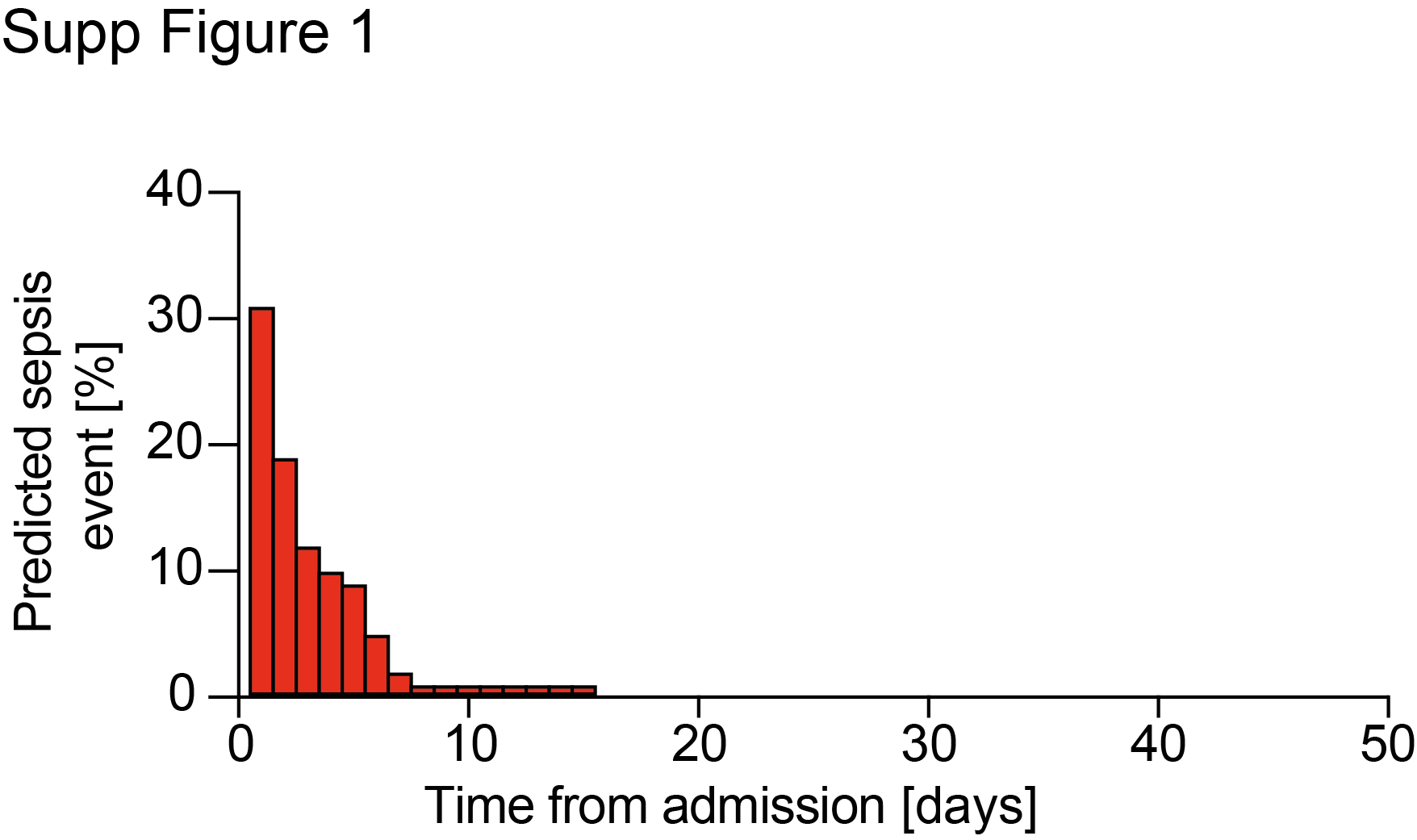


**Supplementary Figure 2. HERACLES Flag distribution according to hospital admission day.**


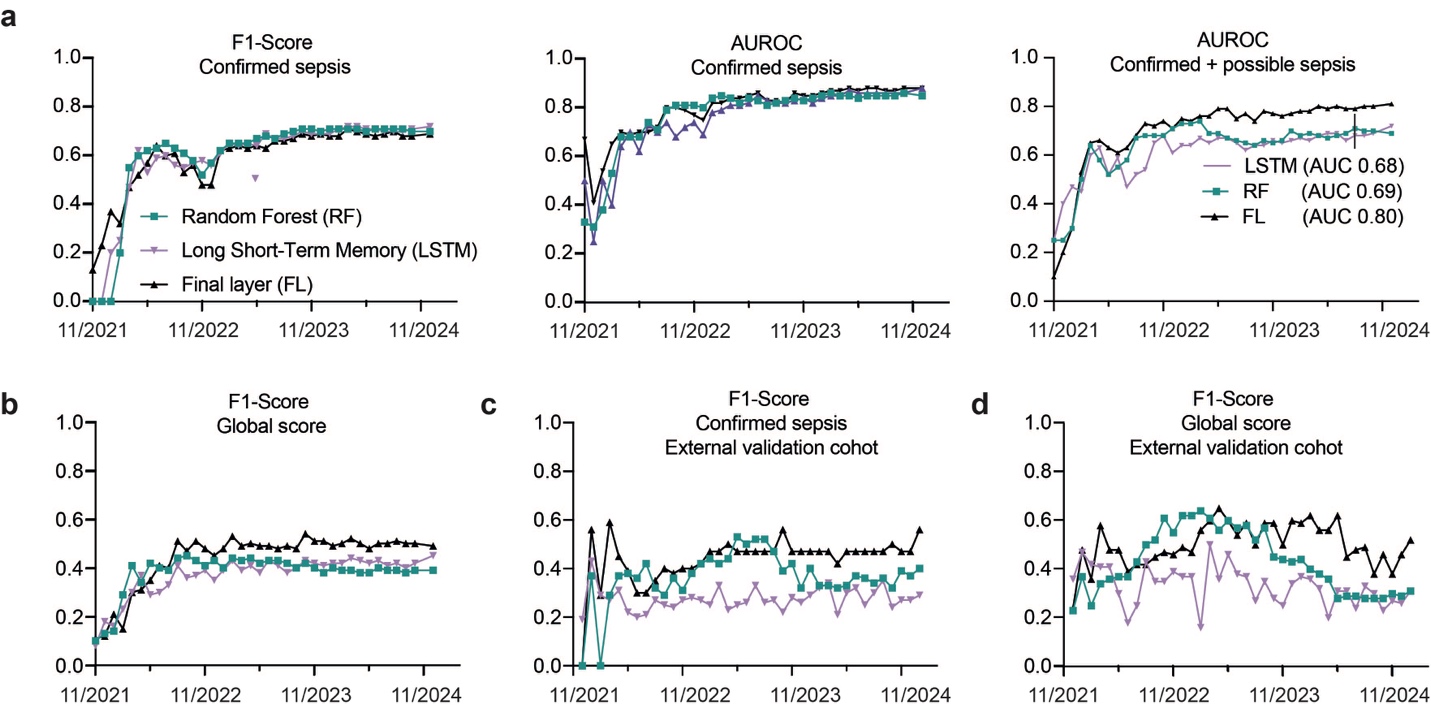


**Supplementary Figure 3. Overview and validation of HERACLES performance.** Representation against time of the performance of HERACLES on internal validation and external validations. Internal validation of HERACLES for confirmed sepsis labels only (a) and for general performance (b), as well as external validation on the isolation ward test set for confirmed sepsis cases (c) and general performance (d) recognition as expressed in F1-score for Random-Forest (teal), Long Short-Term Memory (lavender) and final layer (black) algorithm over time.


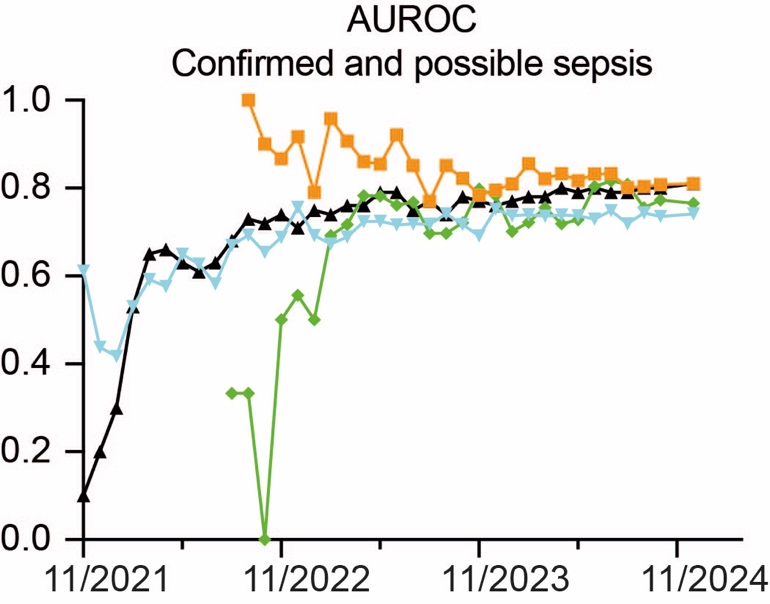


**Supplementary Figure 4. HERACLES submodel AUROC for possible and confirmed sepsis.** Overall performance by AUROC overtime for context-specific models - IE training for GIS, green, MED, blue, and EMD, orange using department-specific events, compared to overall performance (black).

**8. Abbreviations**

AI Artificial Intelligence

AUROC Area Under the Receiver Operating Characteristic

CDC Center of Diseases Control

CHUV Centre Hospitalier Universitaire Vaudois/Lausanne University Hospital

CI Confidence Interval

CVD Cardiovascular Department

EMD Emergency Department

EHR Electronic Health Records

EPFL Ecole Polytechnique Federale de Lausanne

ETL Extract-Transform-Load

FAIR Findability, Accessibility, Interoperability, and Reuse

GIS Gastrointestinal Surgery

HERACLES Health Evaluation and Risk Assessment for Clinical Early Sepsis

ICD International Classification of Diseases

ICU Intensive Care Units

ID Infectious Diseases

IM Internal Medicine

IRB Institutional Review Board

IT Information technology

LAD Locomotor Apparatus Department

LHS Learning Health System

LOINC Logical Observation Identifiers Names & Codes

LSTM Long Short-Term Memory

MED MEdicine Department

NEWS National Early Warning Score

NSD Neurosciences Department

RF Random Forest

SCP Sepsis Care Program

SDSC Swiss Data Science Center

SNOMED_CT Systematized Nomenclature of Medicine - Clinical Terms

SOFA Sequential Organ Failure Assessment

SPHN Swiss Personalized Health Network

SSC Surviving Sepsis Campaign

SLHS Sepsis Learning Health System

OD Oncology Department

OLS Ordinary Least Squares

OR Odds Ratio

1. [↑](#footnote-ref-1)
